# Supplementary material for: TBK1‐mediated phosphorylation of LC3C and GABARAP‐L2 controls autophagosome shedding by ATG4 protease
Source: EMBO Rep. 2019 Nov 11;21(1):e48317. doi: 10.15252/embr.201948317 (PMC6945063; doi:10.15252/embr.201948317)
Supplement: Supplementary file 1 — Expanded View Figures PDF [file EMBR-21-e48317-s001.pdf]

# Expanded View Figures

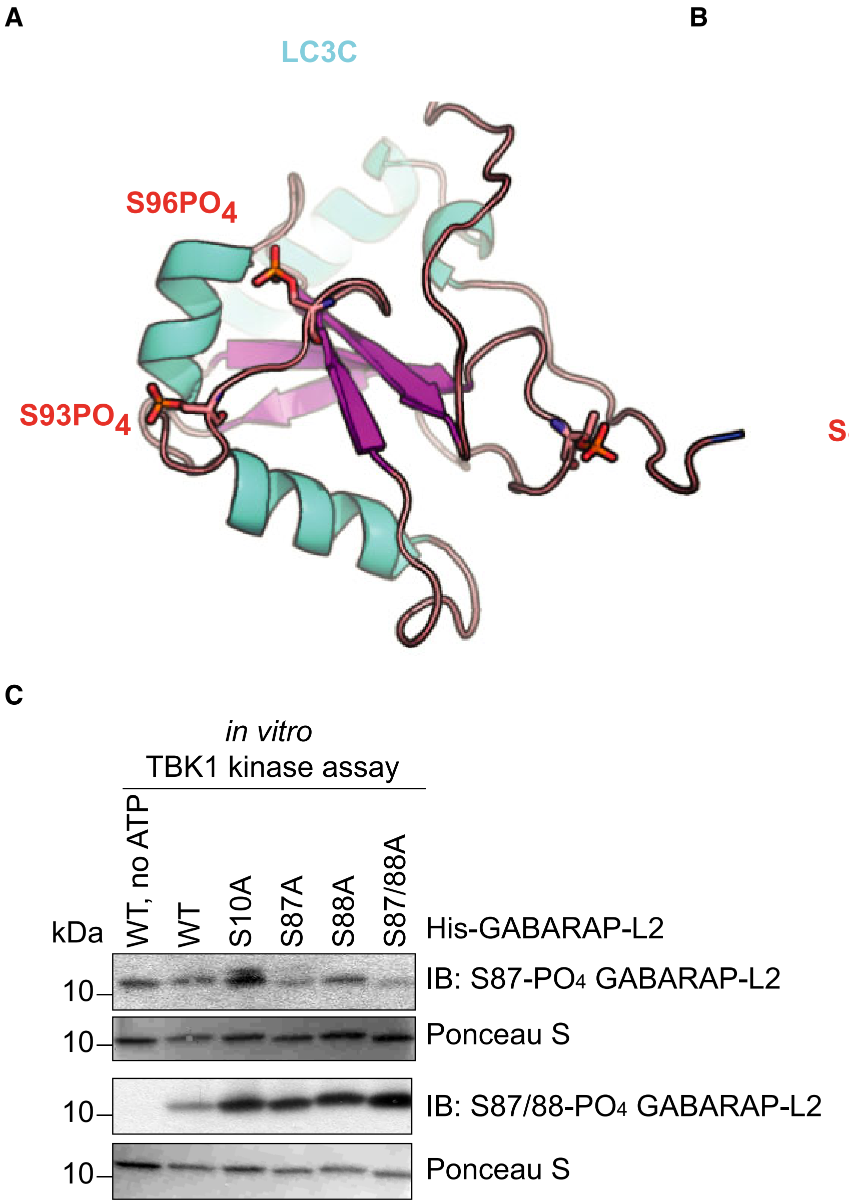


**Figure EV1. Phosphorylation sites of GABARAP-L2 and LC3C.**

A Structure of $LC3C_{8-125}$ (PDB: 3WAM) with modeled phosphate groups (red sticks) at S93 and S96.

B Topologically equivalent positions in GABARAP-L2 (PDB: 4CO7), S87 and S88, are also phosphorylated (red sticks) by TBK1. Phosphorylation sites are on the opposite face of the LIR binding pocket of LC3 proteins.

C SDS–PAGE and Western blot of *in vitro* TBK1 kinase assay with His-GABARAP-L2 WT or mutants as substrates to test the respective phospho-GABARAP-L2 antibodies for their specificity.

Source data are available online for this figure.

© 2019 The Authors 

**Figure EV2. Phosphorylation of S93 and S96 of LC3C affects ATGB binding.**

A SDS–PAGE and Western blot of HEK293T cell lysates and GFP immunoprecipitations. Cells were transfected with Flag-tagged ATG4A, ATG4B, ATG4C, or ATG4D and GFP-tagged LC3C or GABARAP-L2 WT or mutants and lysates used for GFP IPs. S93/96D mutation of LC3C and S87/88D mutation of GABARAP-L2 impede binding to ATG4A, ATG4B, ATG4C, and ATG4D.

B Bio-Layer Interferometry measurement of GST-ATG4A binding to His-GABARAP-L2 WT and S87/88D.

C–E (C) WT LC3C-ATG4B complex (D) with additional LIR interactions and (E) with phosphorylated LC3C residues (S93 and S96) subjected to MD simulations and binding free energy computations using MM-PBSA (see Materials and Methods) approach. A residue-wise decomposition of the total binding free energy mapped onto the LC3C structure displays locally favorable (blue), neutral (white), and unfavorable (red) residue interaction with ATG4B (gray surface). S93 and S96 positions in WT complexes contribute favorably (blue), whereas in the phosphorylated complex they contribute unfavorably (red) toward complex formation. The thickness of the backbone scales linearly with the binding energy of LC3C-ATG4B complexes.

Source data are available online for this figure.


© 2019 The Authors

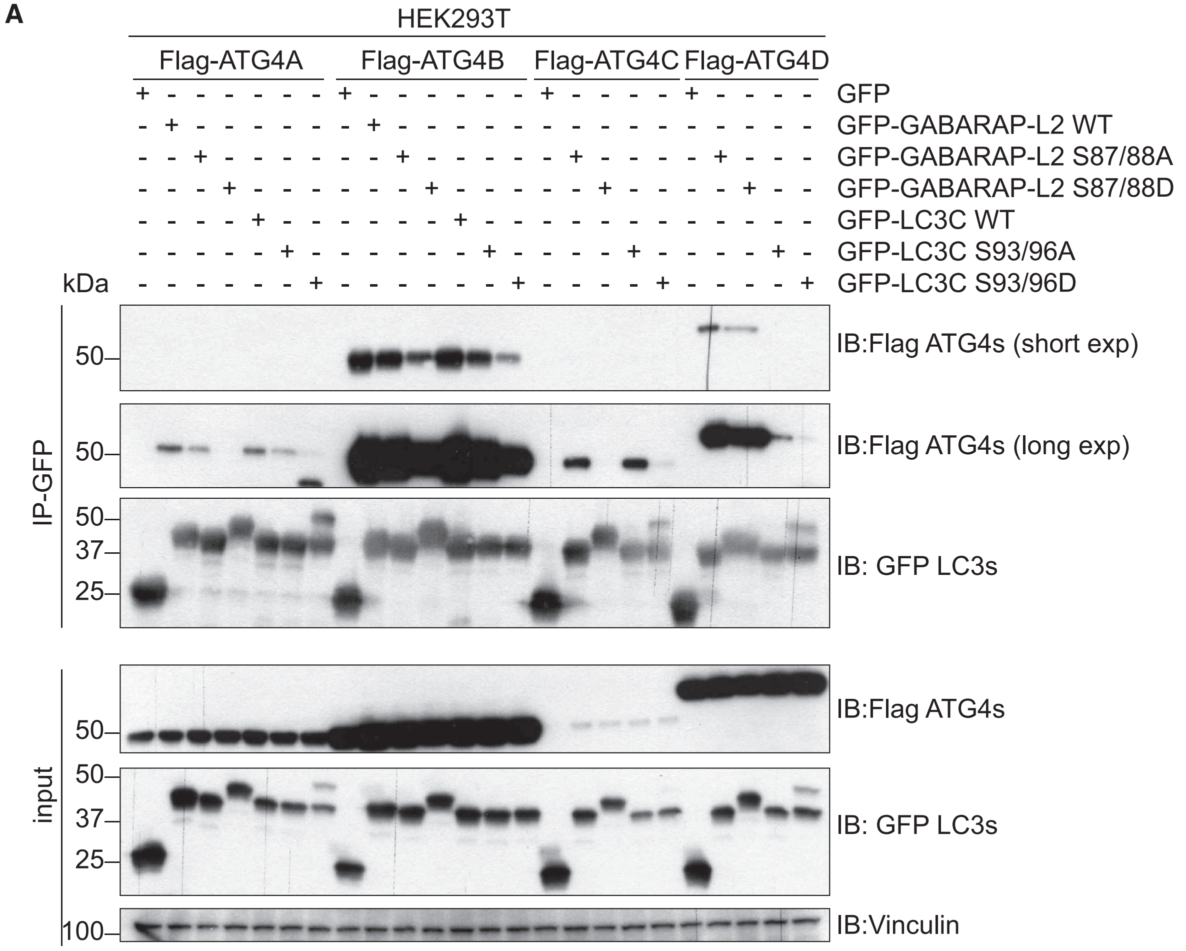


**B**

| ATG4A binding | $K_d$ | $R^2$ | $Chi^2$ |
|---|---|---|---|
| GABARAP-L2 WT | 9.9 μM | 0.99 | 0 |
| GABARAP-L2 S87/88D | 24 μM | 0.98 | 0.0001 |

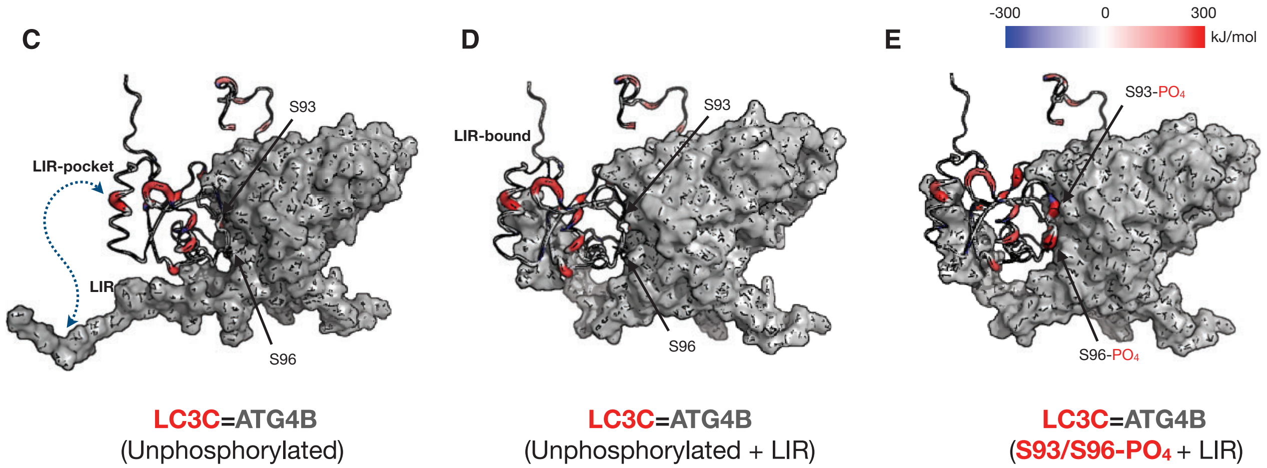


**Figure EV2.**

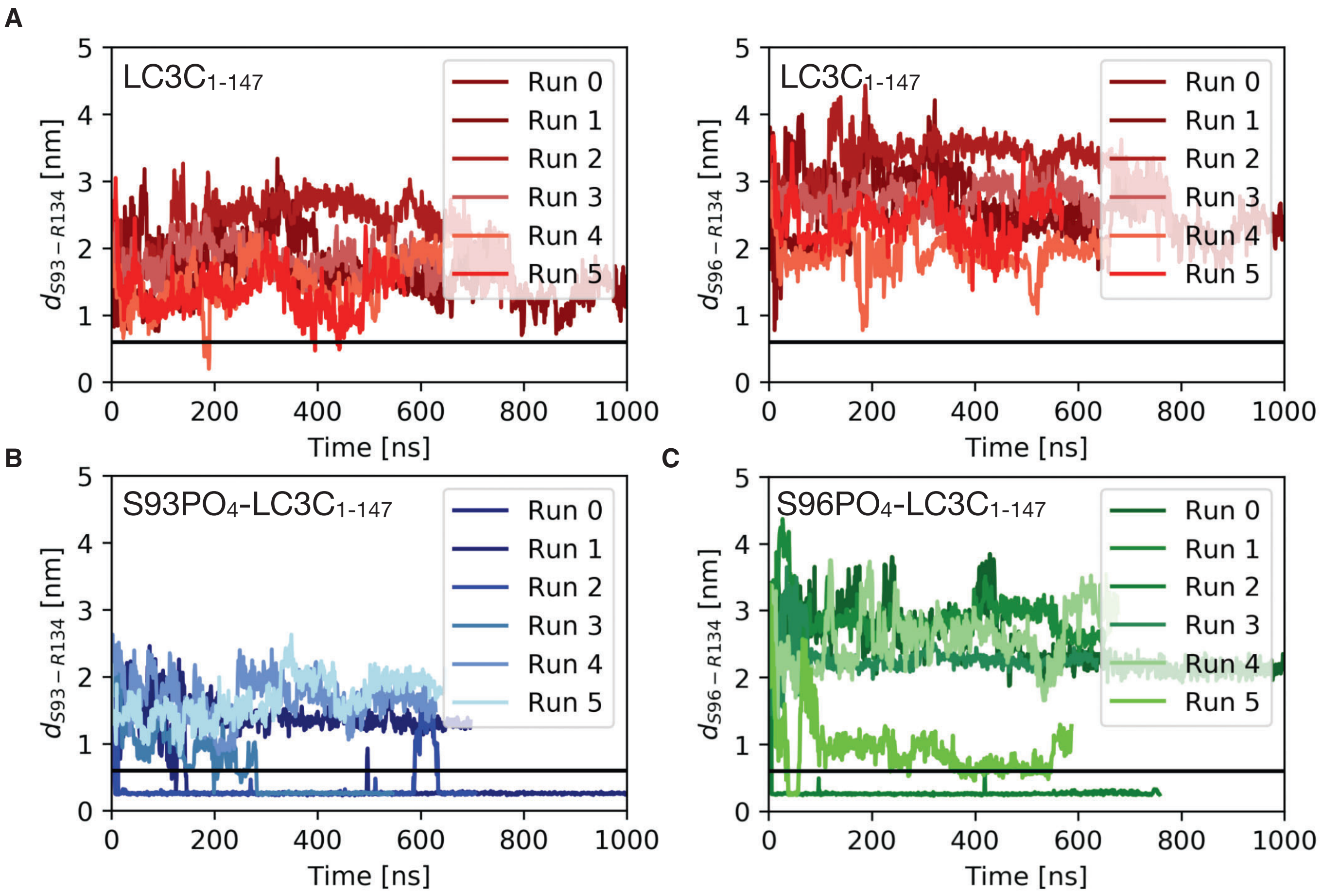


**Figure EV3. Phosphorylation at S93 and S96 affects LC3C C-terminal tail structure, thereby impeding ATG4-mediated cleavage.**

A–C Plots of the minimum distance between R134 and S93/S96 report on intramolecular salt-bridge formation in MD simulations of the free LC3C with (A) unphosphorylated S93 and S96, (B) S93-$PO_4$, and (C) S96-$PO_4$. Replicas ($n$ = 6) were initiated with different initial velocities after equilibration (at 310 K, 1 bar, and 150 mM NaCl) to capture variations in the C-terminal tail dynamics in phosphorylated and unphosphorylated LC3C. They consistently show the formation of intramolecular salt bridges between the phosphorylated serine residues and the positively charged R134 in the C-terminal tail. The horizontal black line (0.6 nm) indicates the cut-off distance for stable electrostatic contact interactions.

 © 2019 The Authors

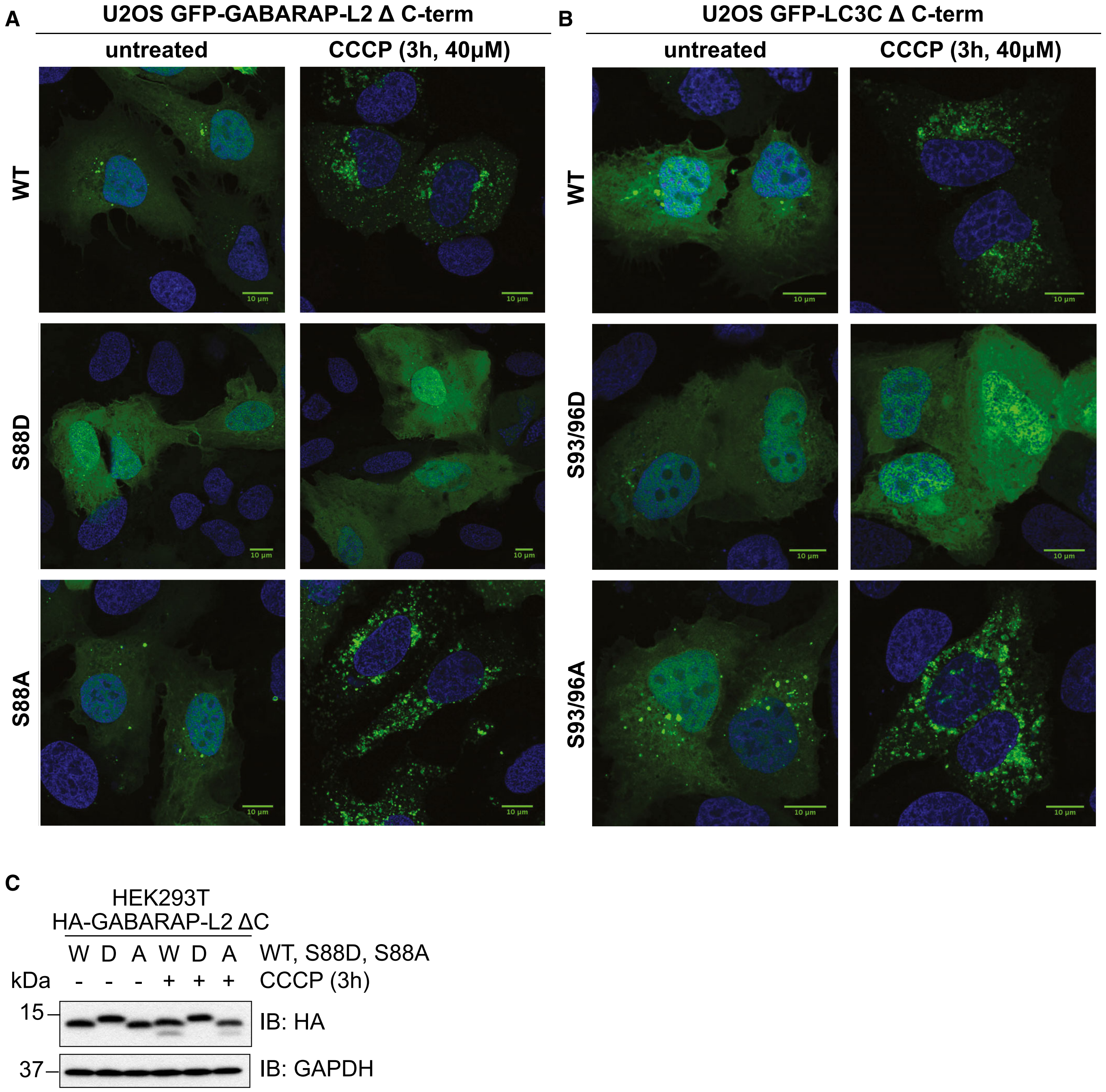


**Figure EV4. Phospho-mimetic Δ C-terminal LC3C or GABARAP-L2 cannot localize to autophagosomes.**

A, B U2OS cells were transfected with GFP-GABARAP-L2 Δ C-terminal (A) or GFP-LC3C Δ C-terminal (B) WT or mutants and HA-Parkin. Mitophagy was induced by the addition of 40 μM CCCP for 3 h. WT, S87/88A Δ C-terminal GABARAP-L2 and WT, S93/96A Δ C-terminal LC3C localize to autophagosomes, whereas S87/88D Δ C-terminal GABARAP-L2 and S93/96D Δ C-terminal LC3C remain dispersed in the cytosol. Scale bar represents 10 μm.

C SDS–PAGE and Western blot of HEK293T cell lysates transfected with HA-GABARAP-L2 Δ C-terminal WT or mutants. Cells were left untreated or treated with 40 μM CCCP for 3 h to induce mitophagy.

Source data are available online for this figure.

© 2019 The Authors

**Figure EV5. TBK1-mediated GABARAP-L2 and LC3C phosphorylation hinders ATG4 de-lipidation and influences autophagy flux.**

A GABARAP-L2 WT- or S87/88D-conjugated liposomes (II) were treated or not with different amounts (2–0.125 μM) of ATG4A, ATG4B, or RavZ for 1 h at 37°C. Samples were then subjected to SDS–PAGE together with unconjugated GABARAP-L2 (I).

B PE-conjugated GABARAP-L2-II WT was incubated in phosphorylation assay buffer in the presence or absence of GST-TBK1 for 4 h and treated or not with 2 μM ATG4A or ATG4B for 30 min. Samples were then subjected to SDS–PAGE together with unconjugated GABARAP-L2-I.

C HeLa cells expressing doxycycline-inducible Parkin and stable mCherry-GFP-LC3C WT or S93/96A were treated with 1 μg/ml doxycycline and 10 μM CCCP for 16 h. mCherry and GFP-positive puncta were assessed by microscopy (Yokogawa CQ1), and the ratio of GFP over mCherry puncta per cell was calculated for each sample. Data are presented as mean ± SD, > 500 cells/condition, $n$ = 3 biological replicates.

Source data are available online for this figure.


© 2019 The Authors

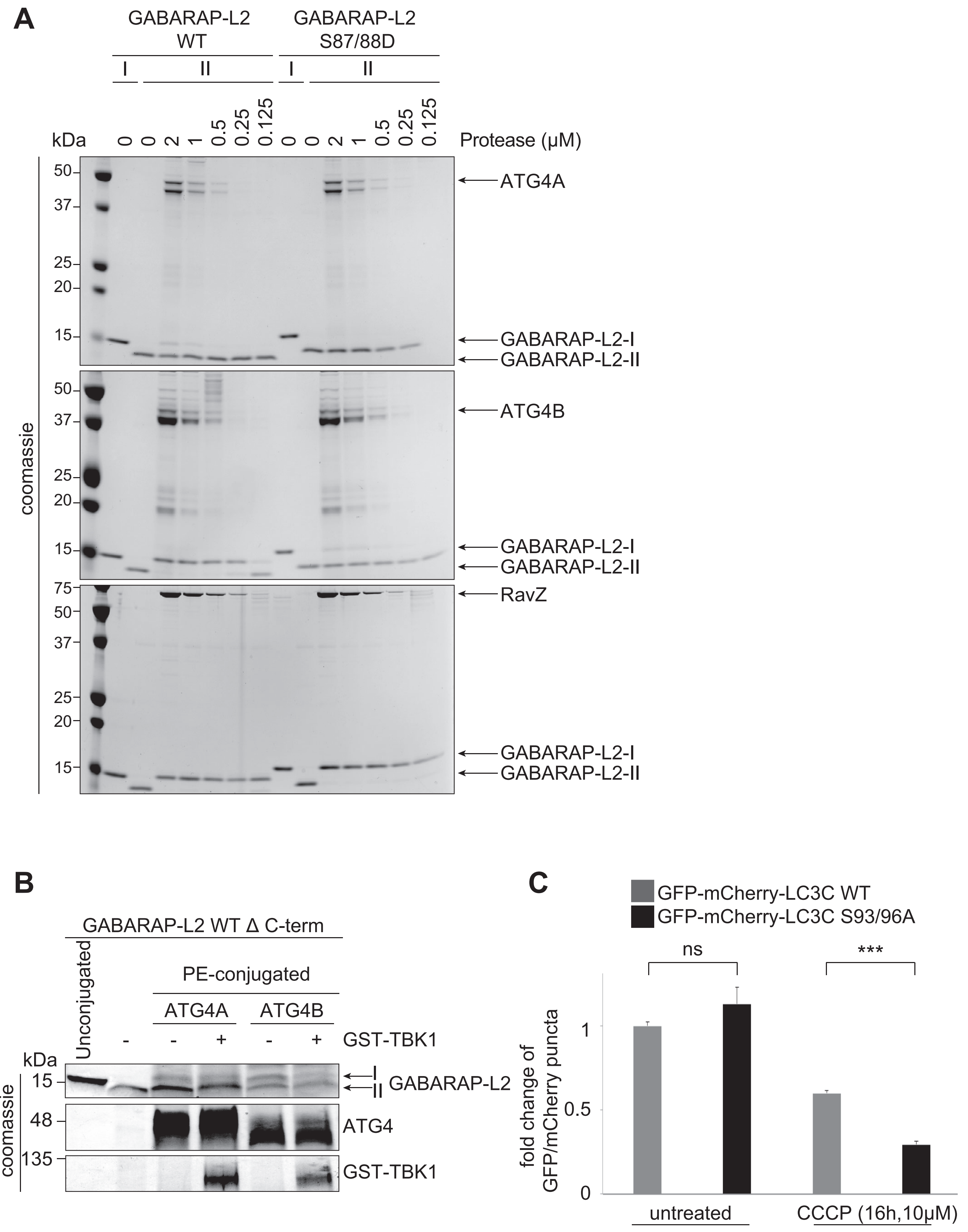
A
GABARAP-L2 WT
GABARAP-L2 S87/88D
I
II
I
II
kDa
0 0 2 1 0.5 0.25 0.125 0 0 2 1 0.5 0.25 0.125
Protease (μM)
50
37
25
20
15
ATG4A
GABARAP-L2-I
GABARAP-L2-II
ATG4B
RavZ
75
coomassie
B
GABARAP-L2 WT Δ C-term
Unconjugated
PE-conjugated
ATG4A
ATG4B
- - + - +
GST-TBK1
kDa
15
48
135
I
II
GABARAP-L2
ATG4
GST-TBK1
coomassie
C
GFP-mCherry-LC3C WT
GFP-mCherry-LC3C S93/96A
ns
***
fold change of GFP/mCherry puncta
1
0.5
0
untreated
CCCP (16h,10μM)

Figure EV5.
