# Supplementary figures and images for: TBK1‐mediated phosphorylation of LC3C and GABARAP‐L2 controls autophagosome shedding by ATG4 protease
Source: EMBO Rep. 2019 Nov 11;21(1):e48317. doi: 10.15252/embr.201948317 (PMC6945063; doi:10.15252/embr.201948317)

Fig EV1C

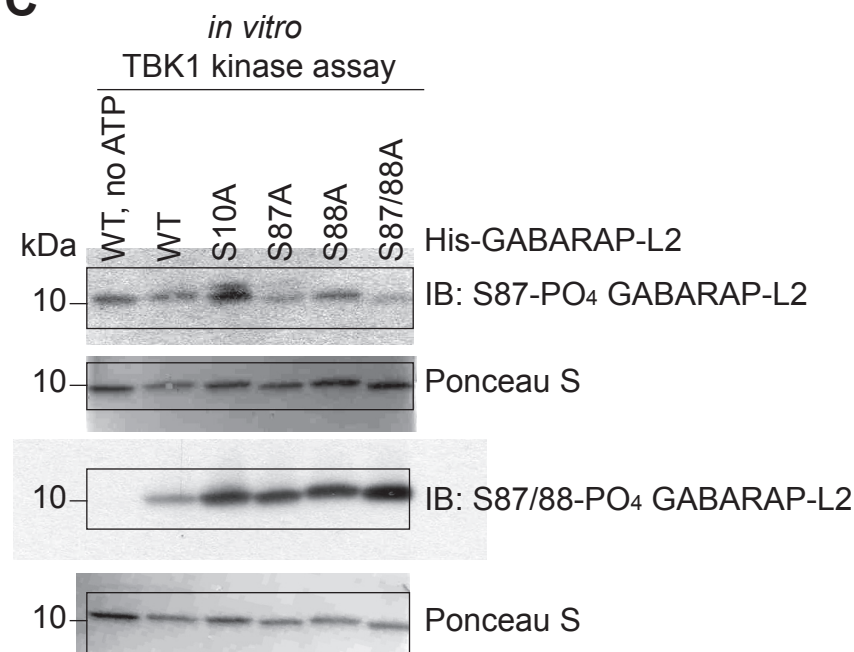

Supplement: Supplementary file 7 — Source Data for Expanded View [file EMBR-21-e48317-s014.zip › Source_Data_for_EV_Figures/Source_Data_for_FigEV1.pdf]

Fig EV2A

HEK293T

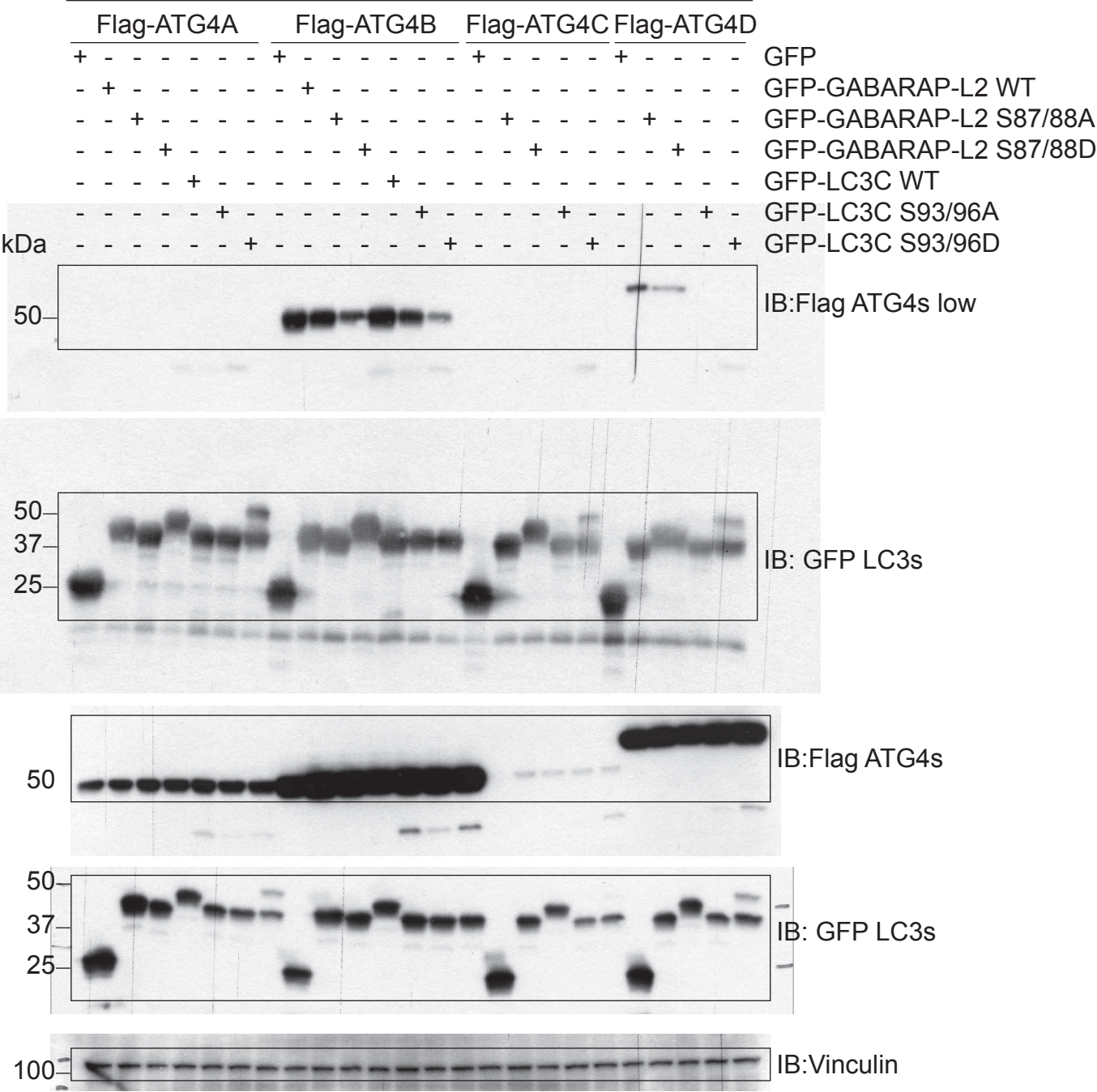

Supplement: Supplementary file 7 — Source Data for Expanded View [file EMBR-21-e48317-s014.zip › Source_Data_for_EV_Figures/Source_Data_for_FigEV2.pdf]

Fig EV 4C

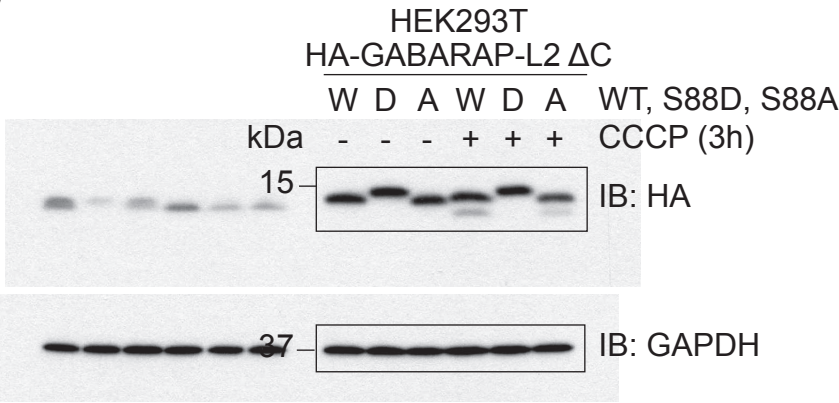

Supplement: Supplementary file 7 — Source Data for Expanded View [file EMBR-21-e48317-s014.zip › Source_Data_for_EV_Figures/Source_Data_for_FigEV4.pdf]

Fig EV5A

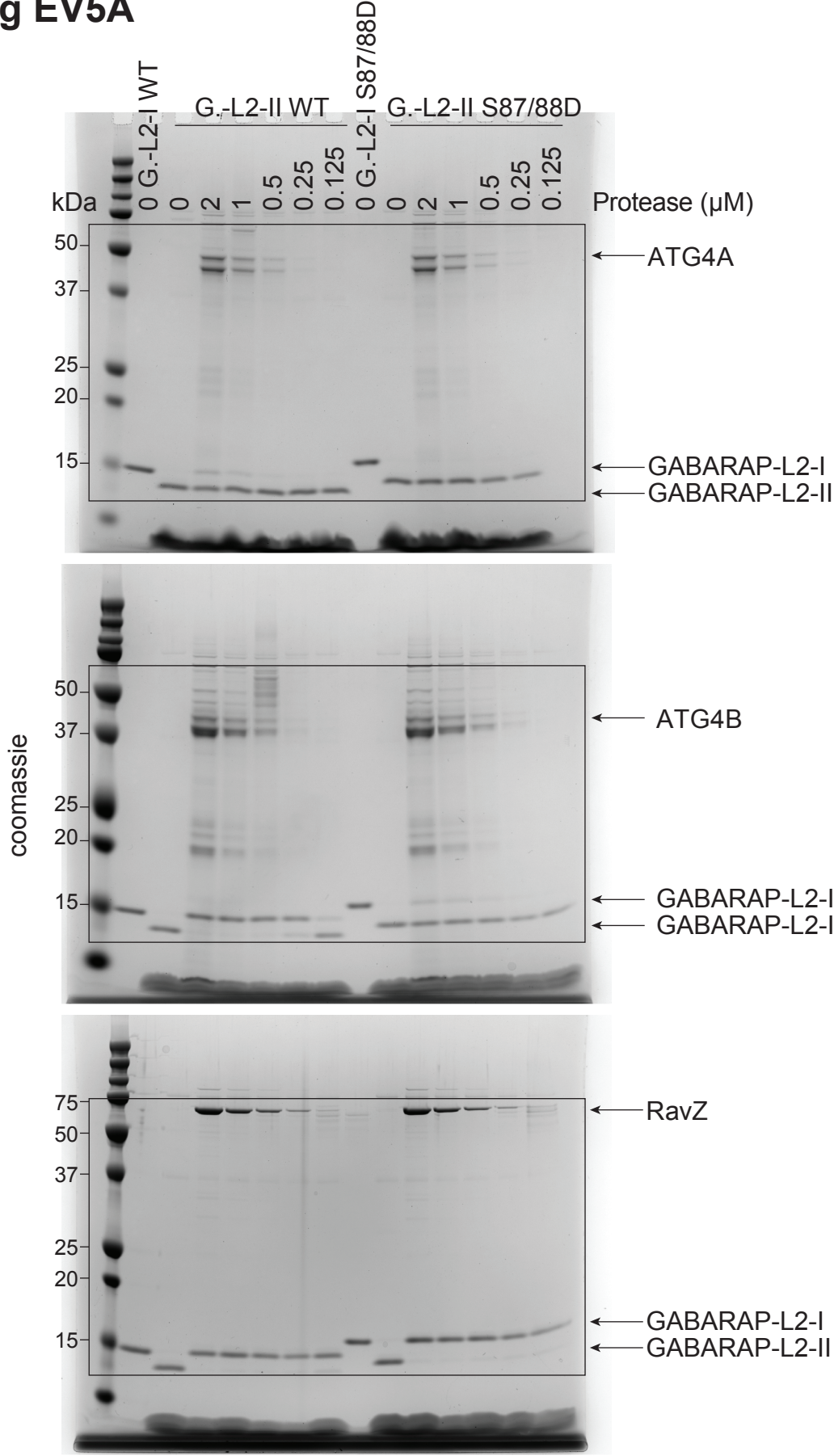

Fig EV5B

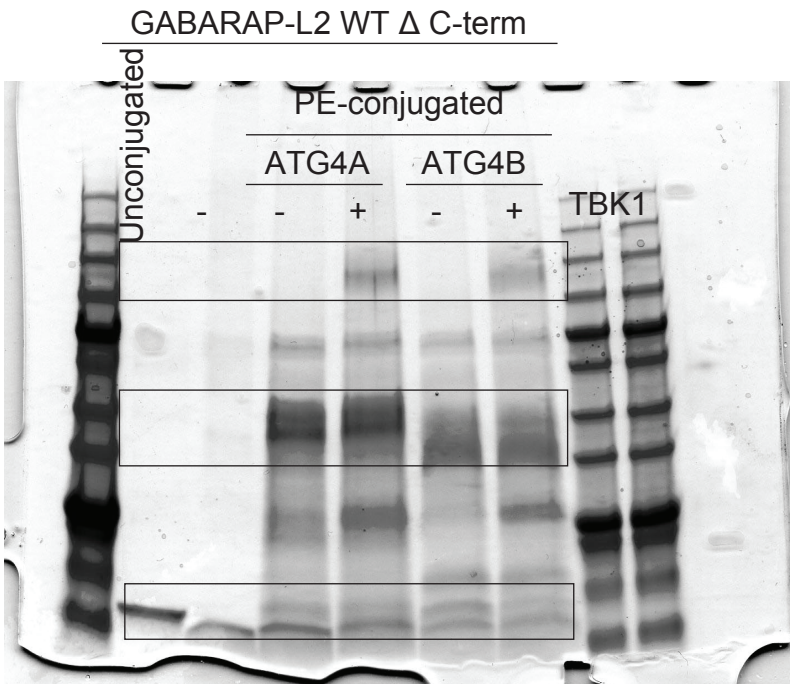

Supplement: Supplementary file 7 — Source Data for Expanded View [file EMBR-21-e48317-s014.zip › Source_Data_for_EV_Figures/Source_Data_for_FigEV5.pdf]

Fig 3A

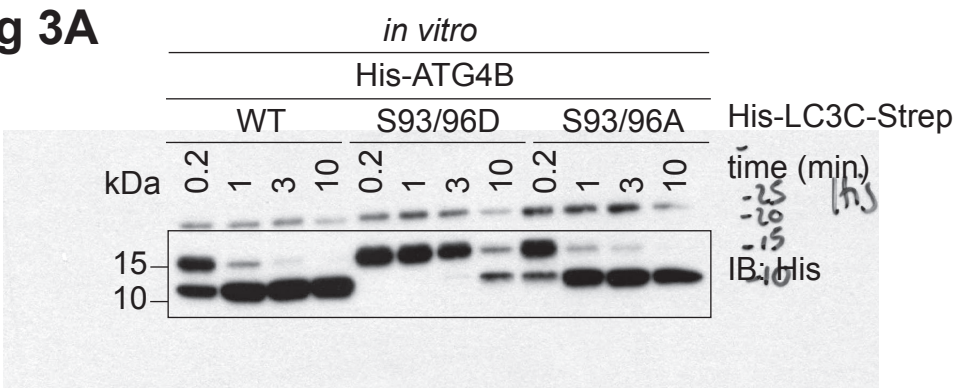

Fig 3B

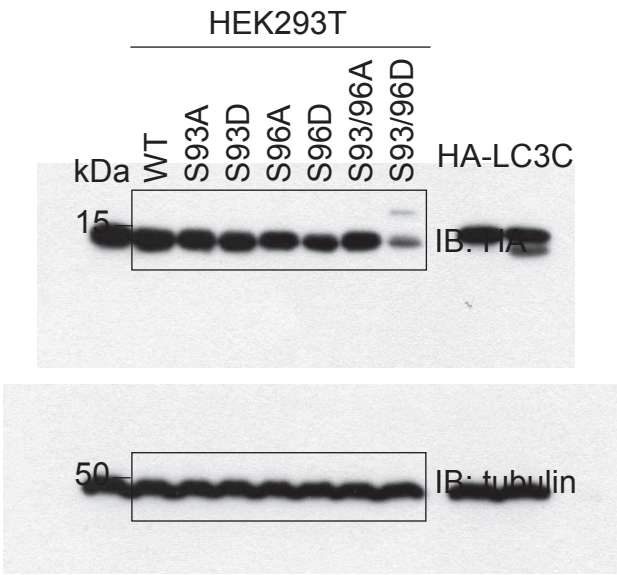

Fig 3C

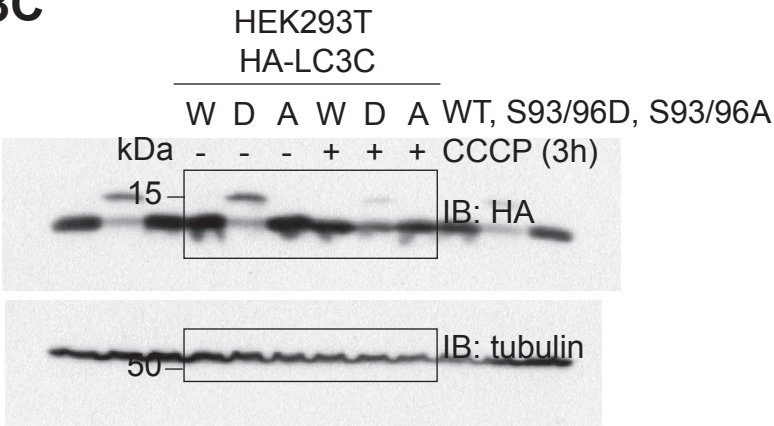

Fig 3D

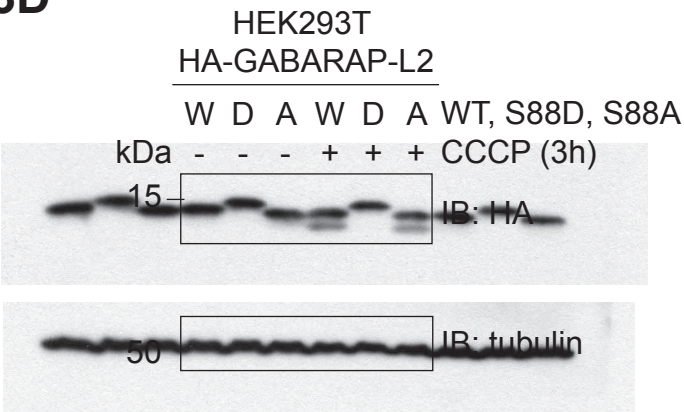

Supplement: Supplementary file 11 — Source Data for Figure 3 [file EMBR-21-e48317-s009.pdf]

Fig 4F

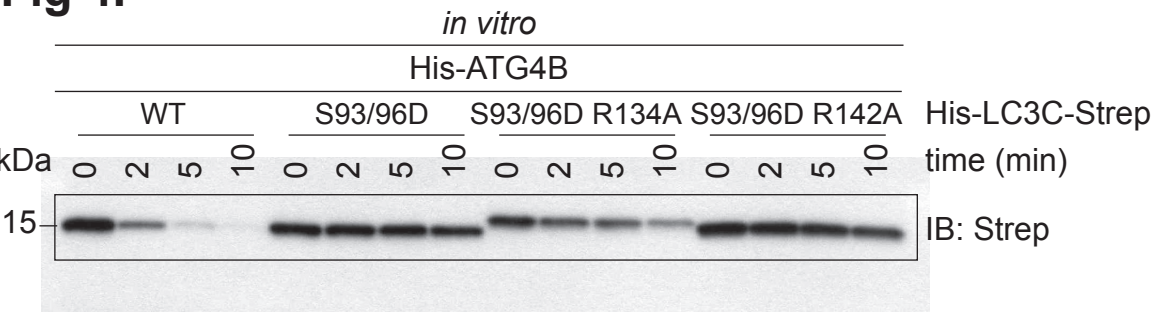

Supplement: Supplementary file 12 — Source Data for Figure 4 [file EMBR-21-e48317-s010.pdf]

Fig 6C

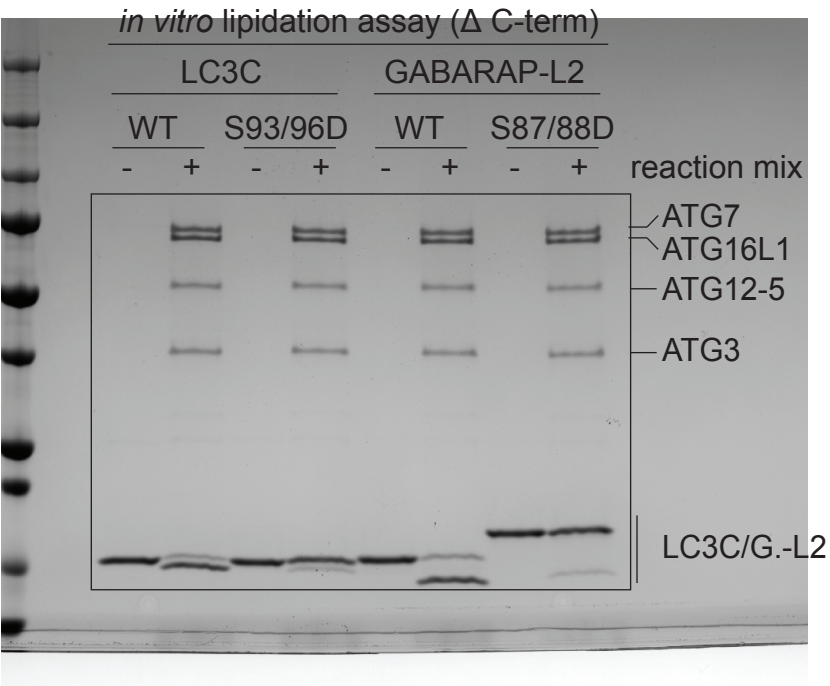

Supplement: Supplementary file 13 — Source Data for Figure 6 [file EMBR-21-e48317-s011.pdf]

Fig 7A

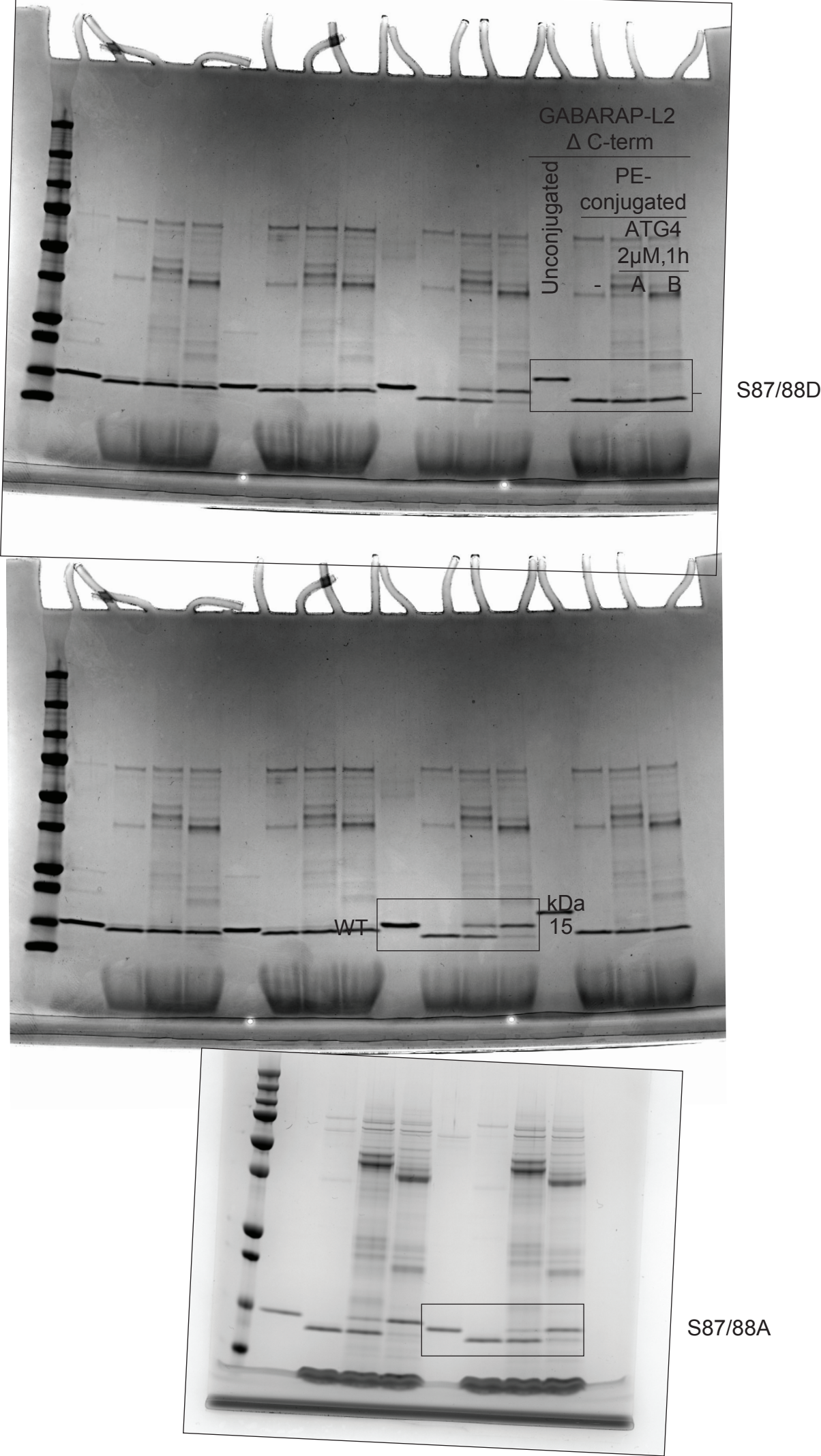

**Fig 7B**

*in vitro* kinase assay  
 $\gamma^{P32}$  ATP, GST-TBK1

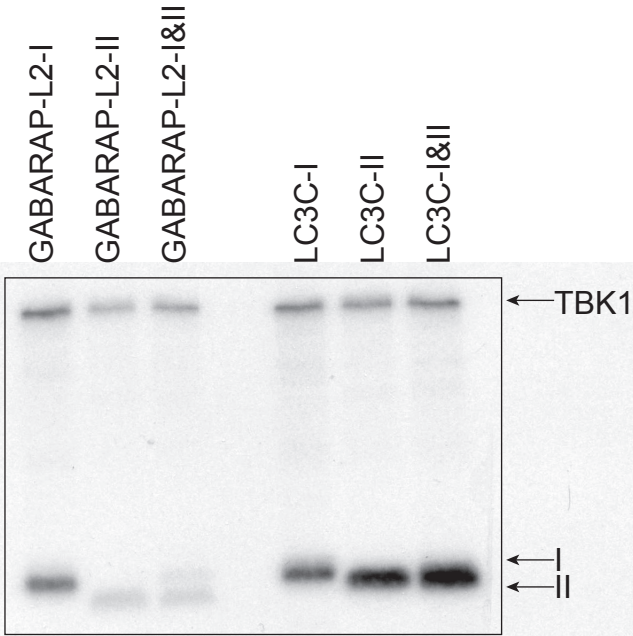

**Fig 7C**

GABARAP-L2 WT  $\Delta$  C-term

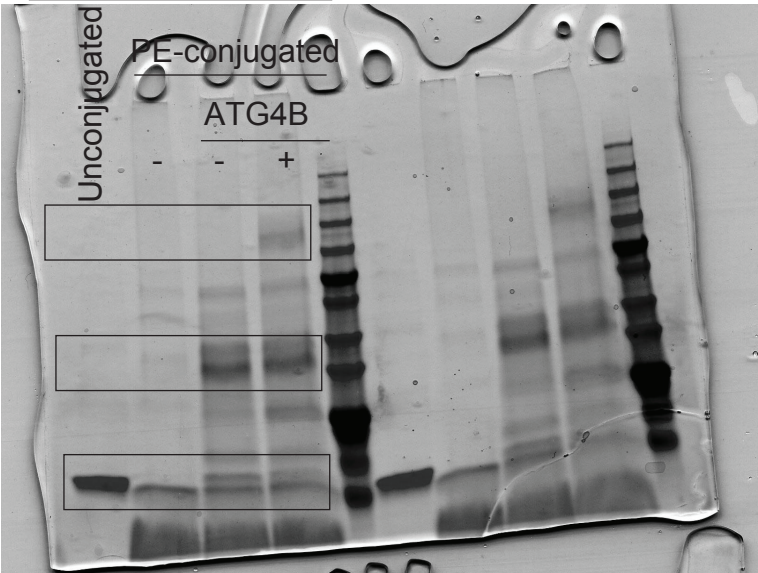

**Fig 7E**

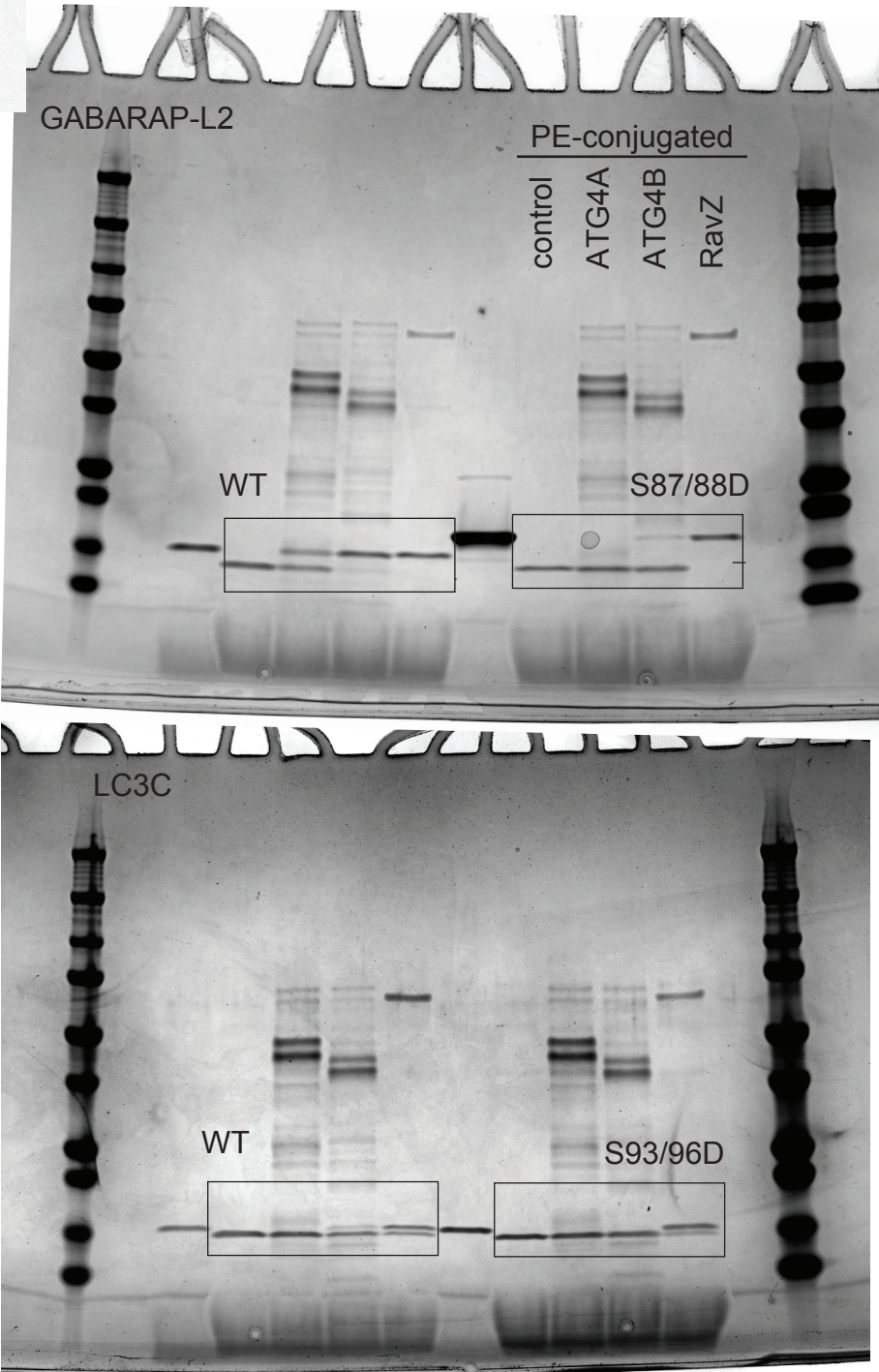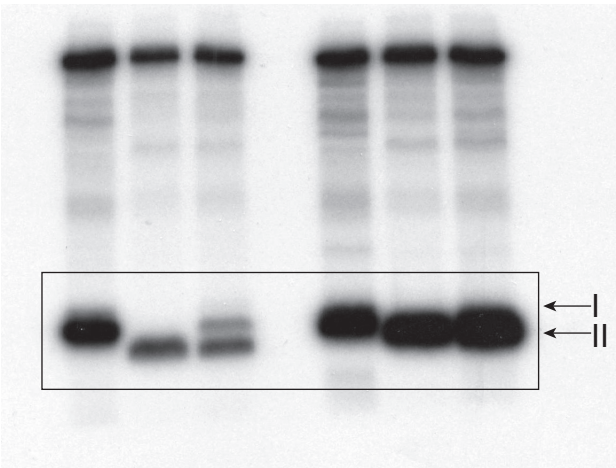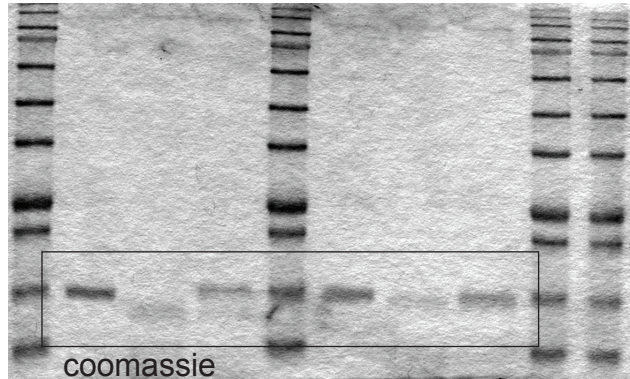

Supplement: Supplementary file 14 — Source Data for Figure 7 [file EMBR-21-e48317-s012.pdf]
