# Supplementary material for: TBK1‐mediated phosphorylation of LC3C and GABARAP‐L2 controls autophagosome shedding by ATG4 protease
Source: EMBO Rep. 2019 Nov 11;21(1):e48317. doi: 10.15252/embr.201948317 (PMC6945063; doi:10.15252/embr.201948317)
Supplement: Supplementary file 9 — Source Data for Figure 1 [file EMBR-21-e48317-s007.pdf]

Fig. 1A

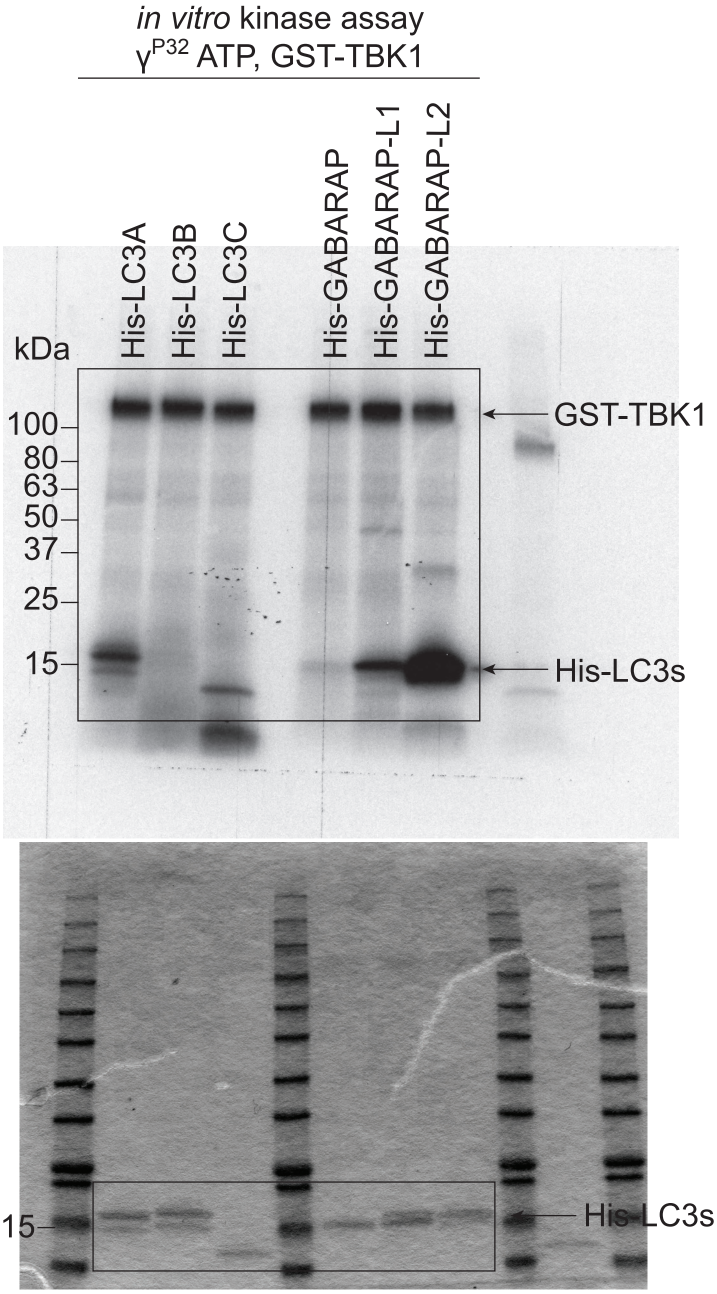

in vitro kinase assay
γP32 ATP, GST-TBK1
His-LC3A
His-LC3B
His-LC3C
His-GABARAP
His-GABARAP-L1
His-GABARAP-L2
kDa
100
80
63
50
37
25
15
GST-TBK1
His-LC3s
15
His-LC3s

Fig 1B

LC3A pS29

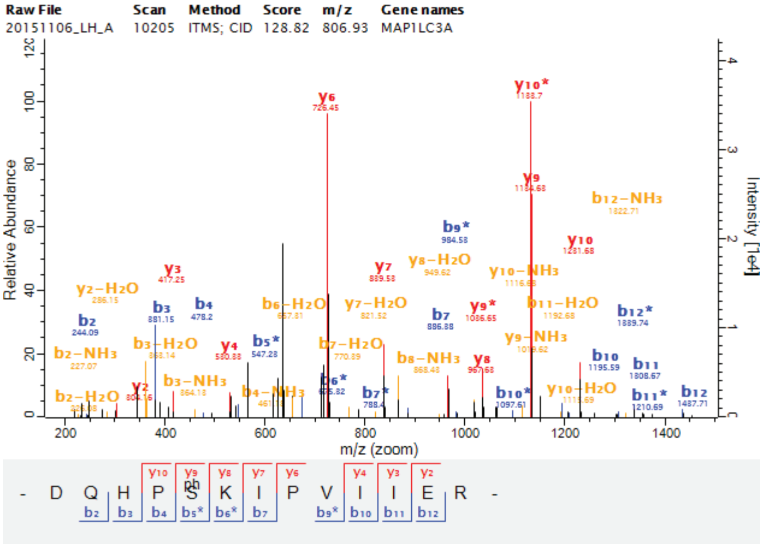

LC3A pS61

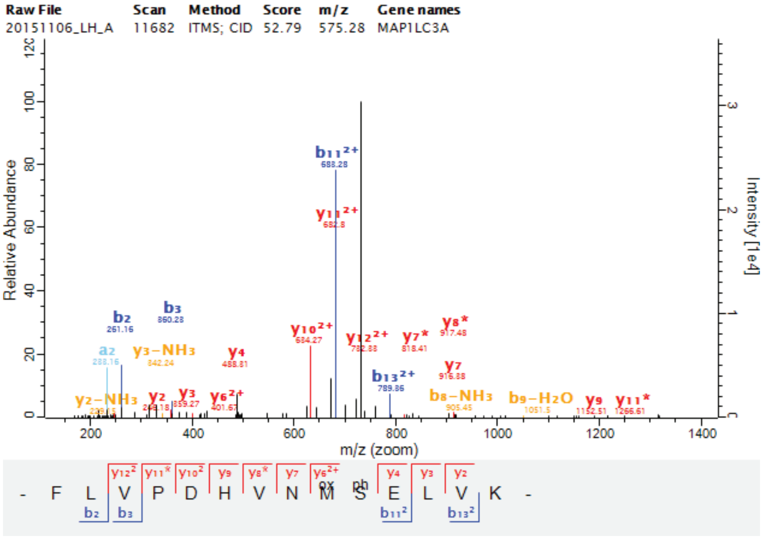

LC3C pT48

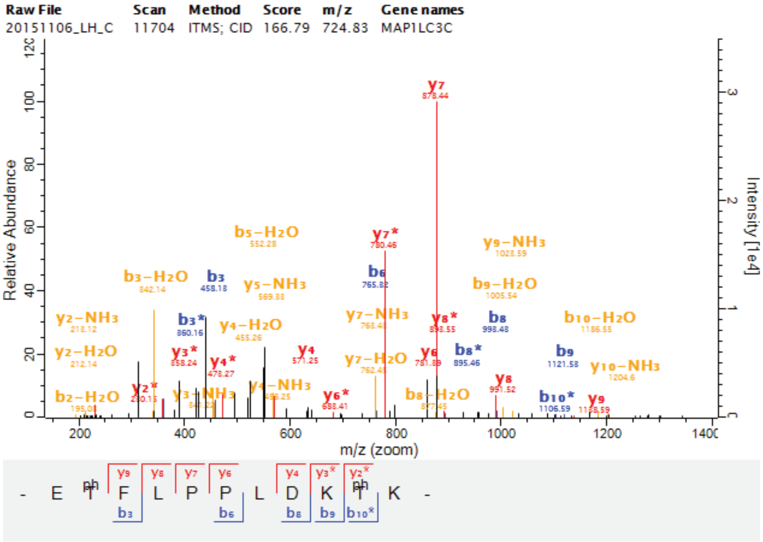

LC3C pT56

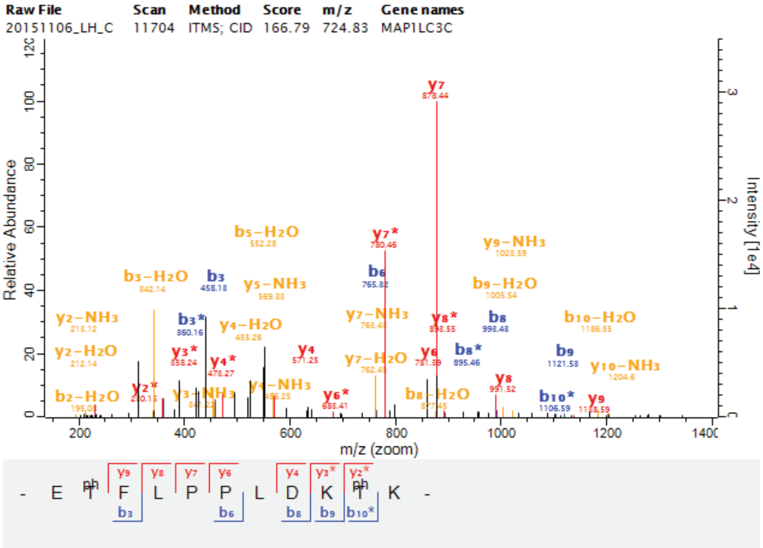

LC3C pS93

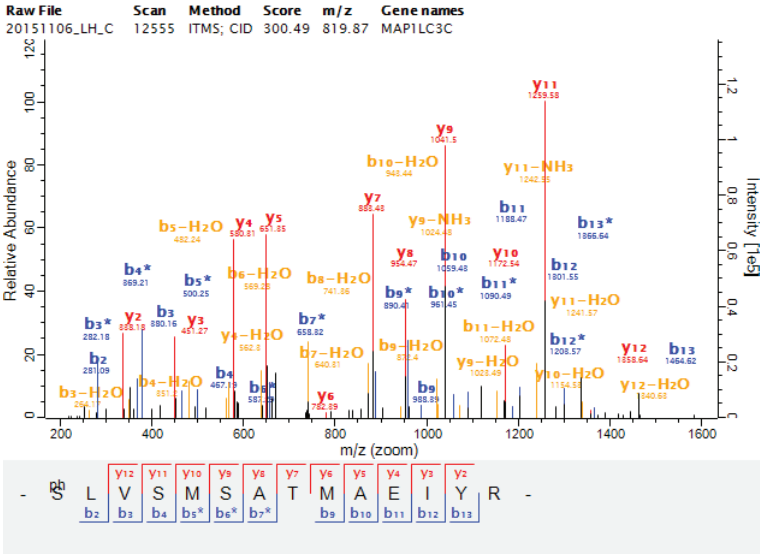

LC3C pS96

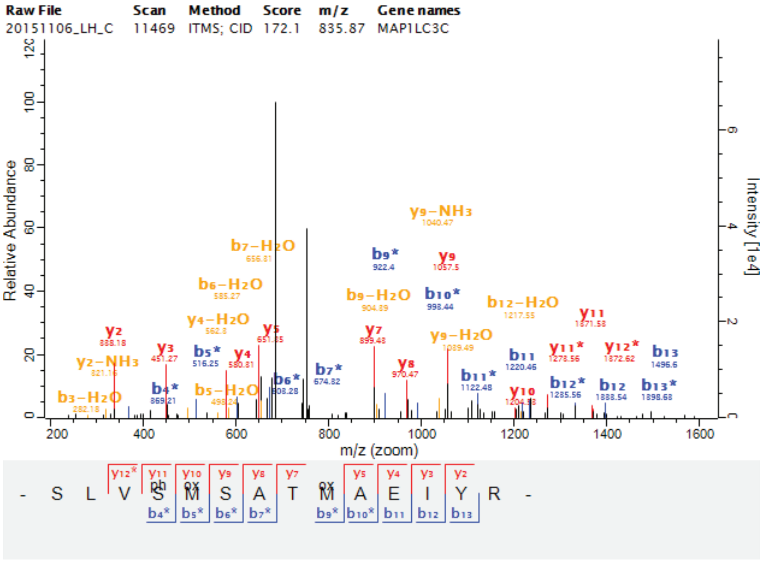

Fig 1B

GABARAP-L1 pY25

Raw File	Scan	Method	Score	m/z	Gene names
20151106_LH_2	12282	ITMS; CID	101.95	697.86	GABARAP;GABARAPL1;GABARAPL2

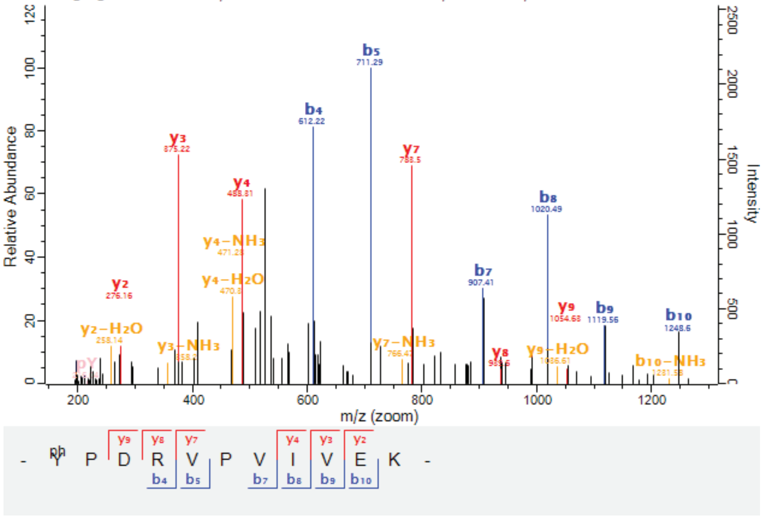

GABARAP-L2 pS10

Raw File	Scan	Method	Score	m/z	Gene names
20151106_LH_2	11201	ITMS; CID	211.82	847.87	GABARAPL2

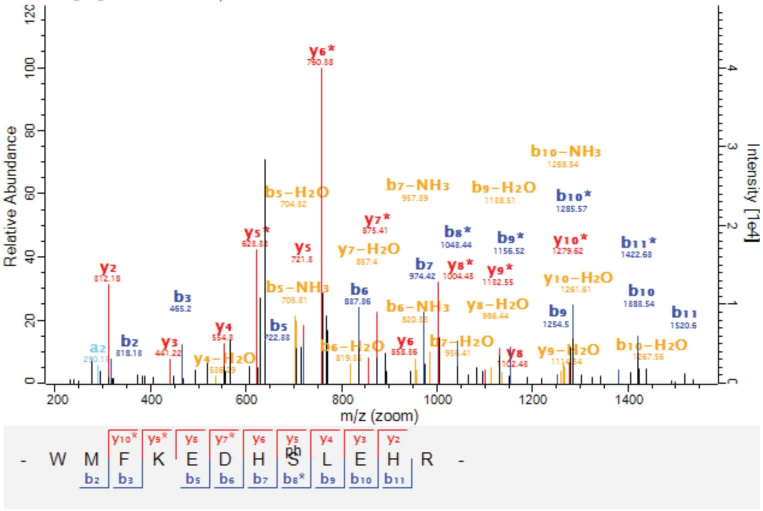

GABARAP-L2 pS39

Raw File	Scan	Method	Score	m/z	Gene names
20151106_LH_2	12659	ITMS; CID	168.93	620.8	GABARAPL2

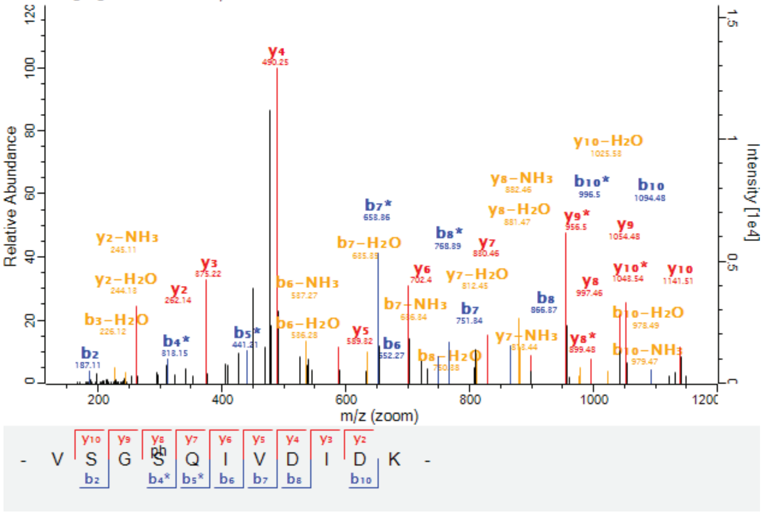

GABARAP-L2 pS87

Raw File	Scan	Method	Score	m/z	Gene names
20151106_LH_2	13275	ITMS; CID	154.81	881.42	GABARAPL2

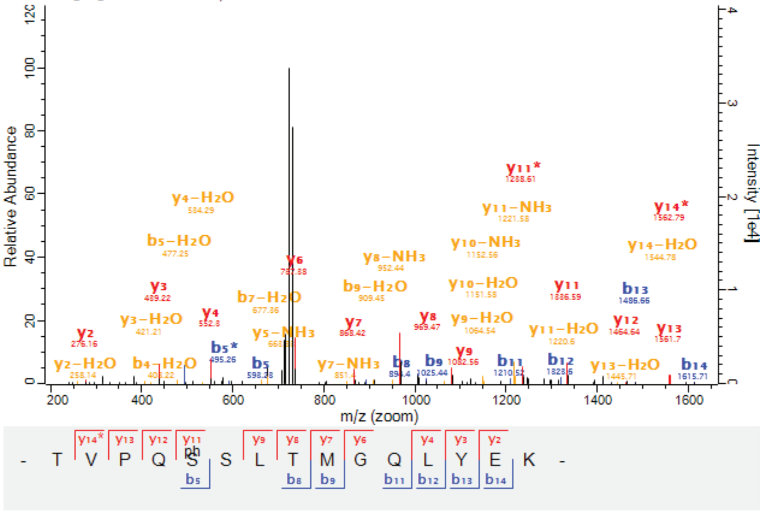

GABARAP-L2 pS88

Raw File	Scan	Method	Score	m/z	Gene names
20151106_LH_2	12226	ITMS; CID	87.82	593.61	GABARAPL2

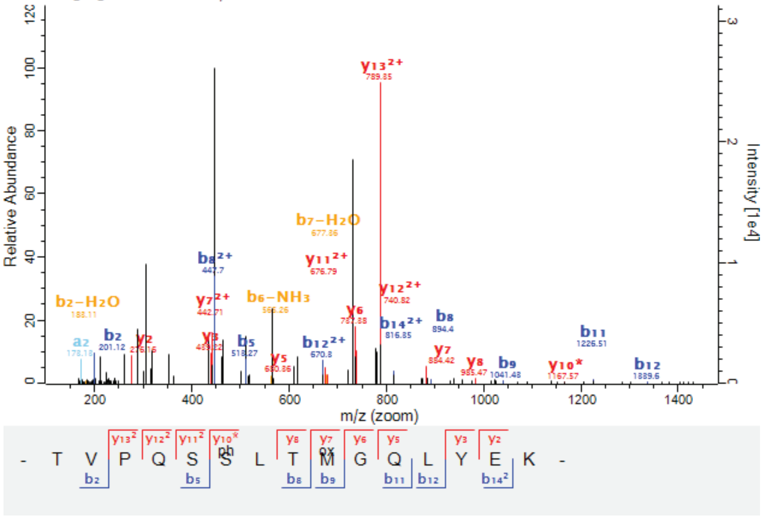

Fig. 1C

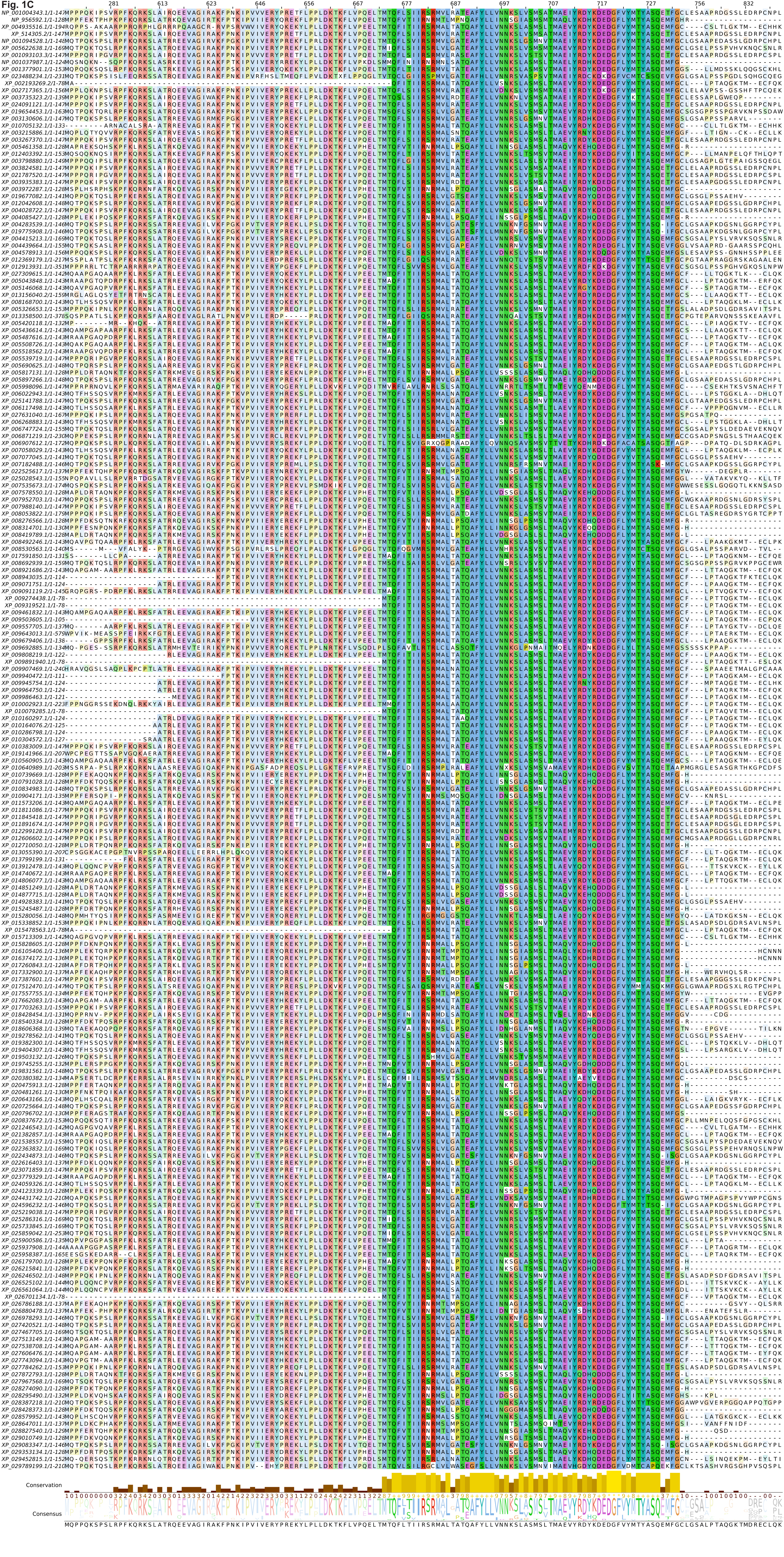
281
613
623
646
656
667
677
687
697
707
717
727
756
832
Conservation
Consensus

Fig. 1D

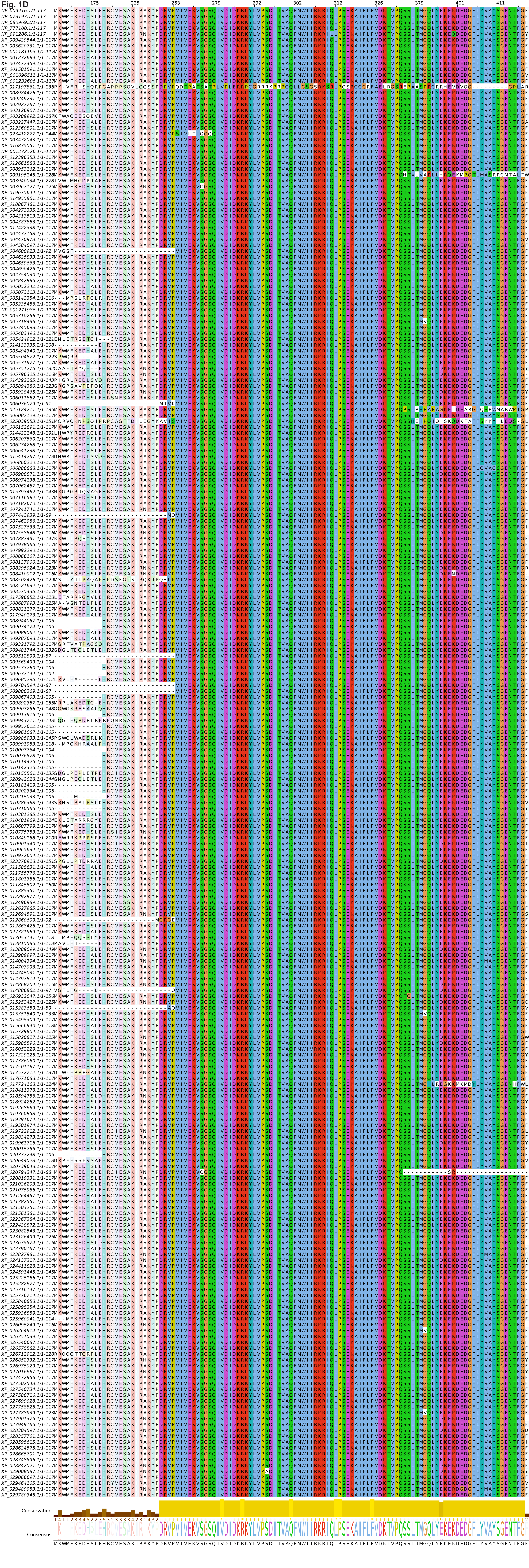
175
225
263
279
292
302
312
326
379
401
411
Conservation
Consensus
