# Supplementary material for: TBK1‐mediated phosphorylation of LC3C and GABARAP‐L2 controls autophagosome shedding by ATG4 protease
Source: EMBO Rep. 2019 Nov 11;21(1):e48317. doi: 10.15252/embr.201948317 (PMC6945063; doi:10.15252/embr.201948317)
Supplement: Supplementary file 10 — Source Data for Figure 2 [file EMBR-21-e48317-s008.pdf]

Fig 2A

GABARAP-L2 pS10

Raw File	Scan	Method	Score	m/z	Gene names
20160115_LH_L2	9248	ITMS; CID	228.17	856.87	GABARAPL2

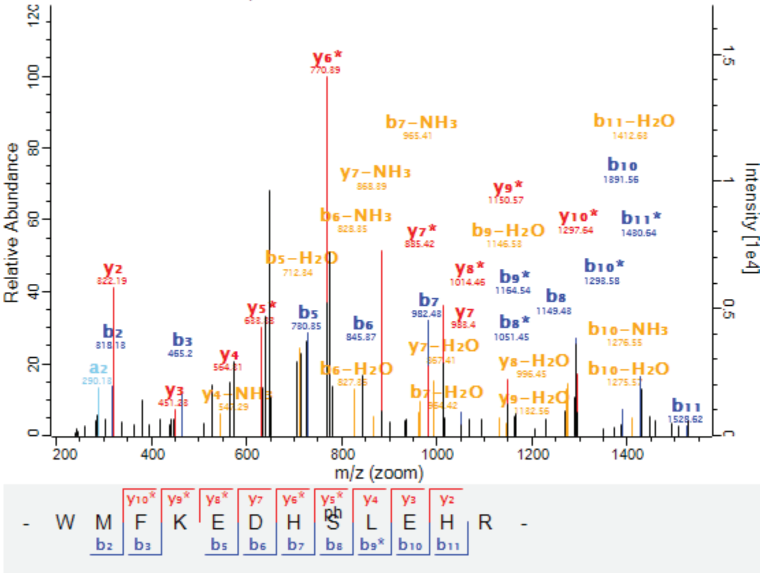

GABARAP-L2 pS39

Raw File	Scan	Method	Score	m/z	Gene names
20160115_LH_L2	11015	ITMS; CID	147.51	624.8	GABARAPL2

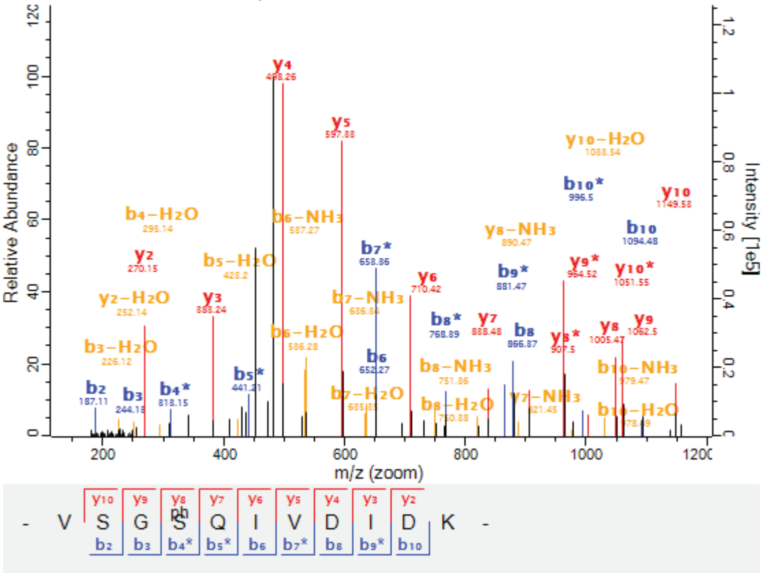

GABARAP-L2 pS87

Raw File	Scan	Method	Score	m/z	Gene names
20160115_LH_L2	10607	ITMS; CID	140.96	889.41	GABARAPL2

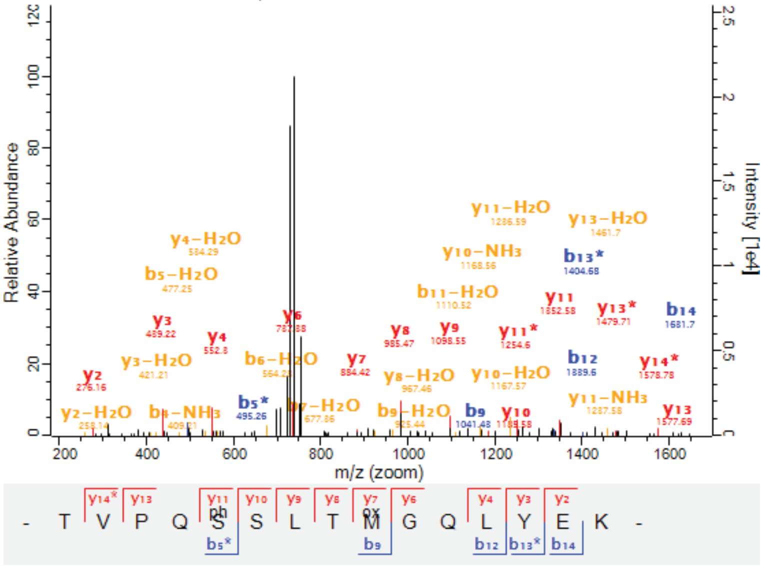

GABARAP-L2 pS88

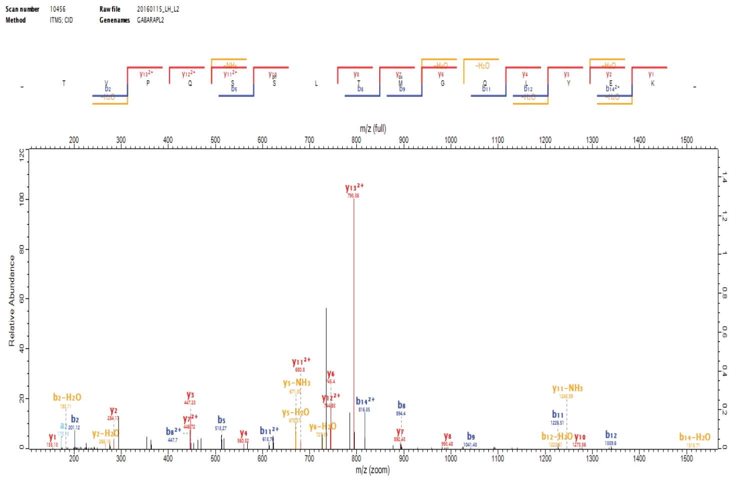

Fig 2A

LC3C pS93

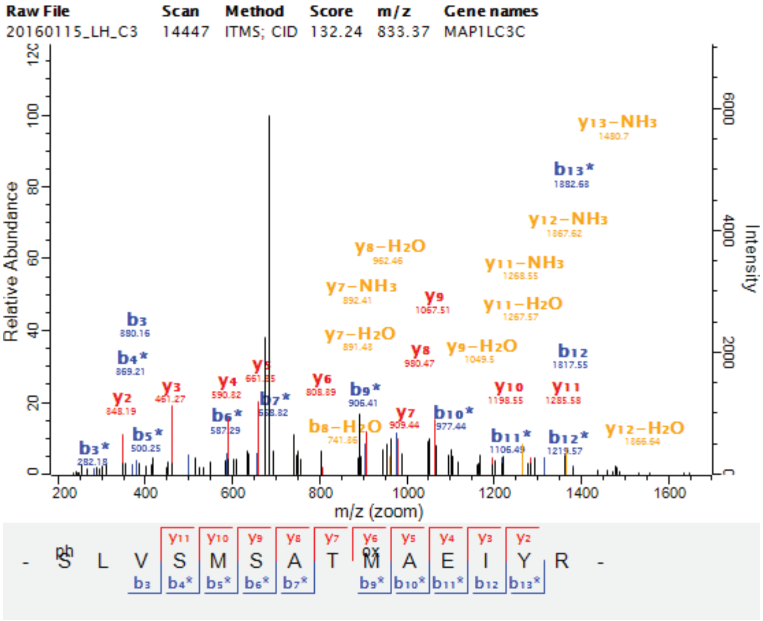

LC3C pS96

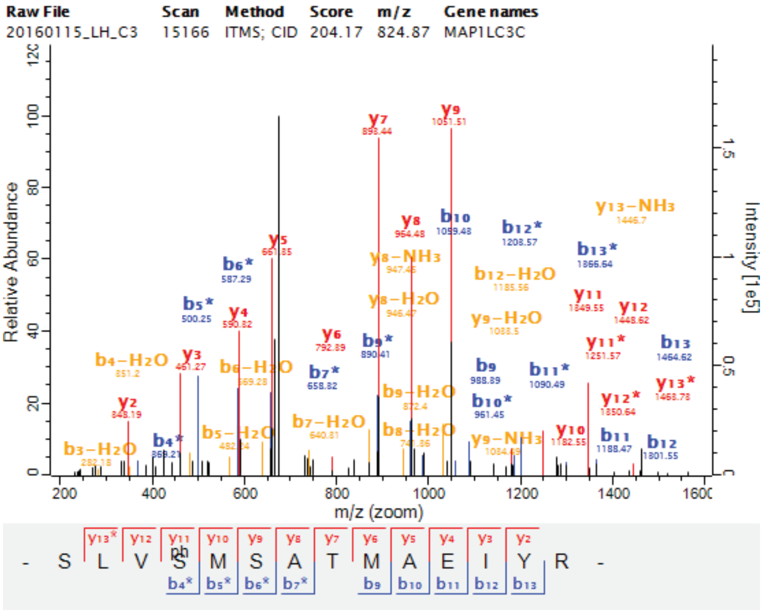

LC3C pS98

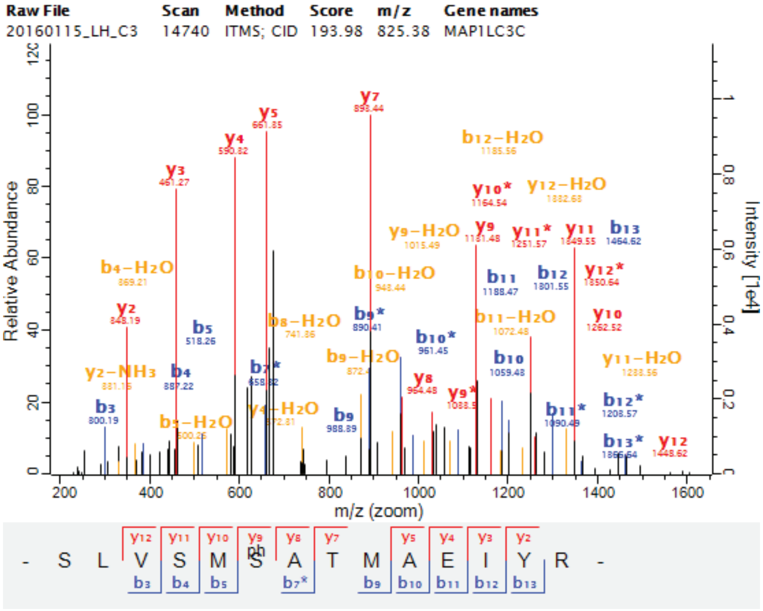

Fig 2B

GABARAP-L2 pS87

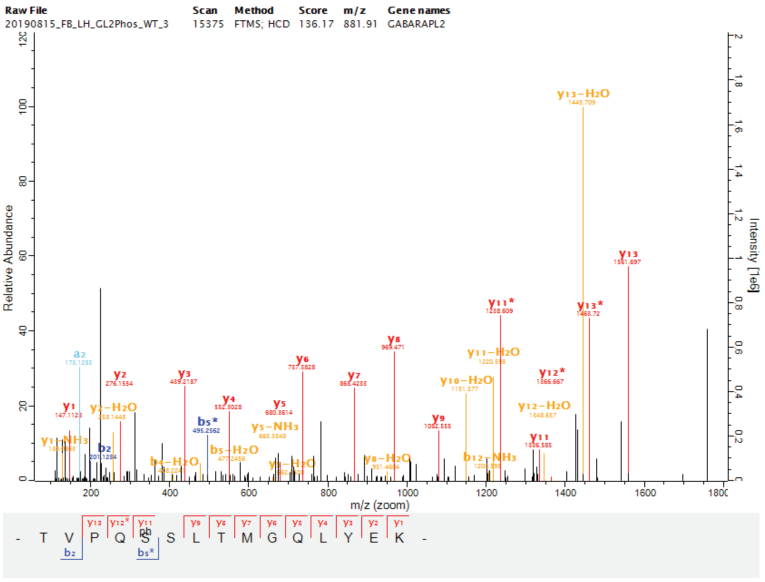

LC3C pS96

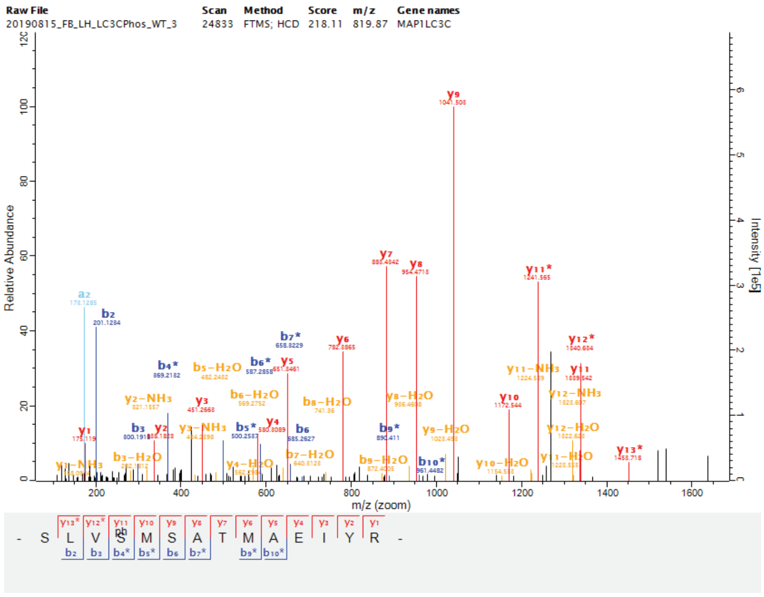

Fig 2C

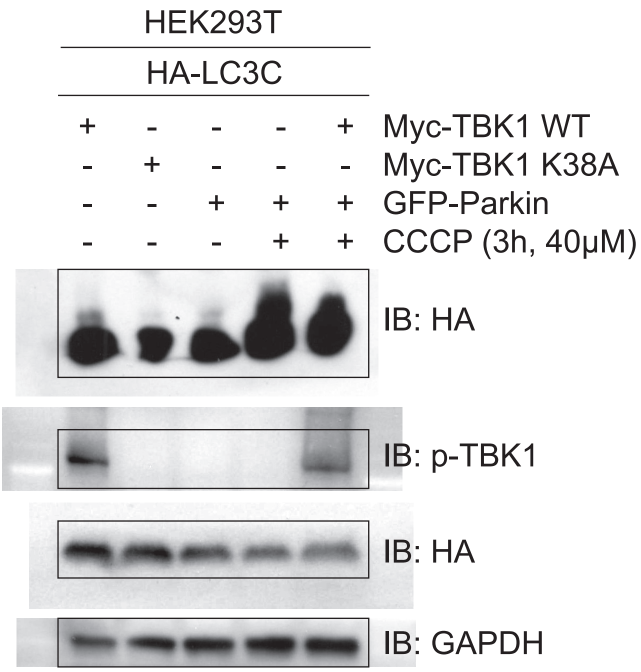

Fig 2D

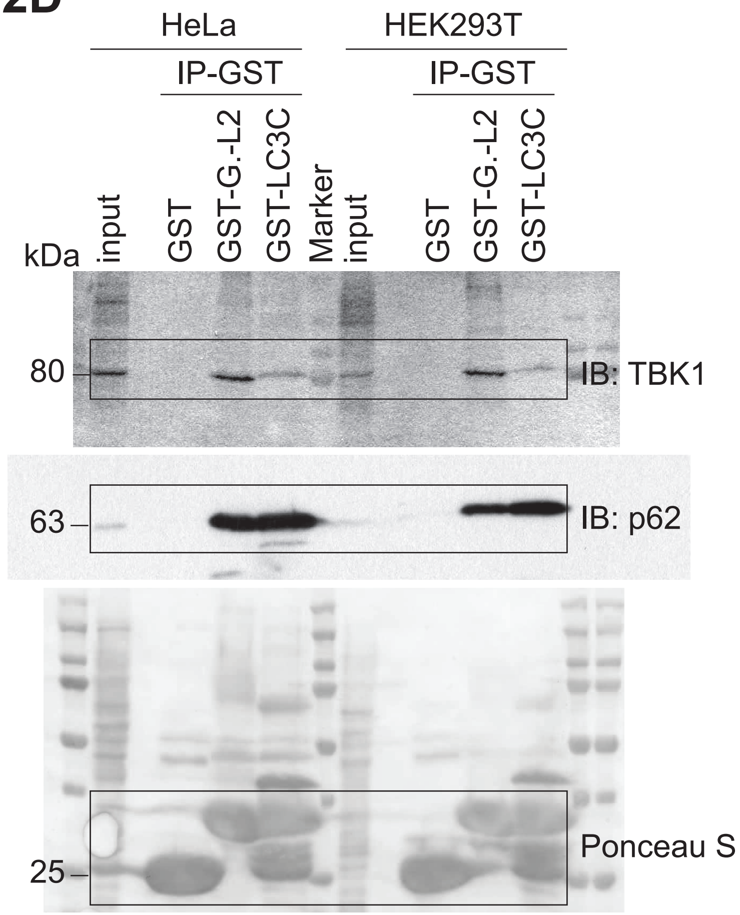

Fig 2E

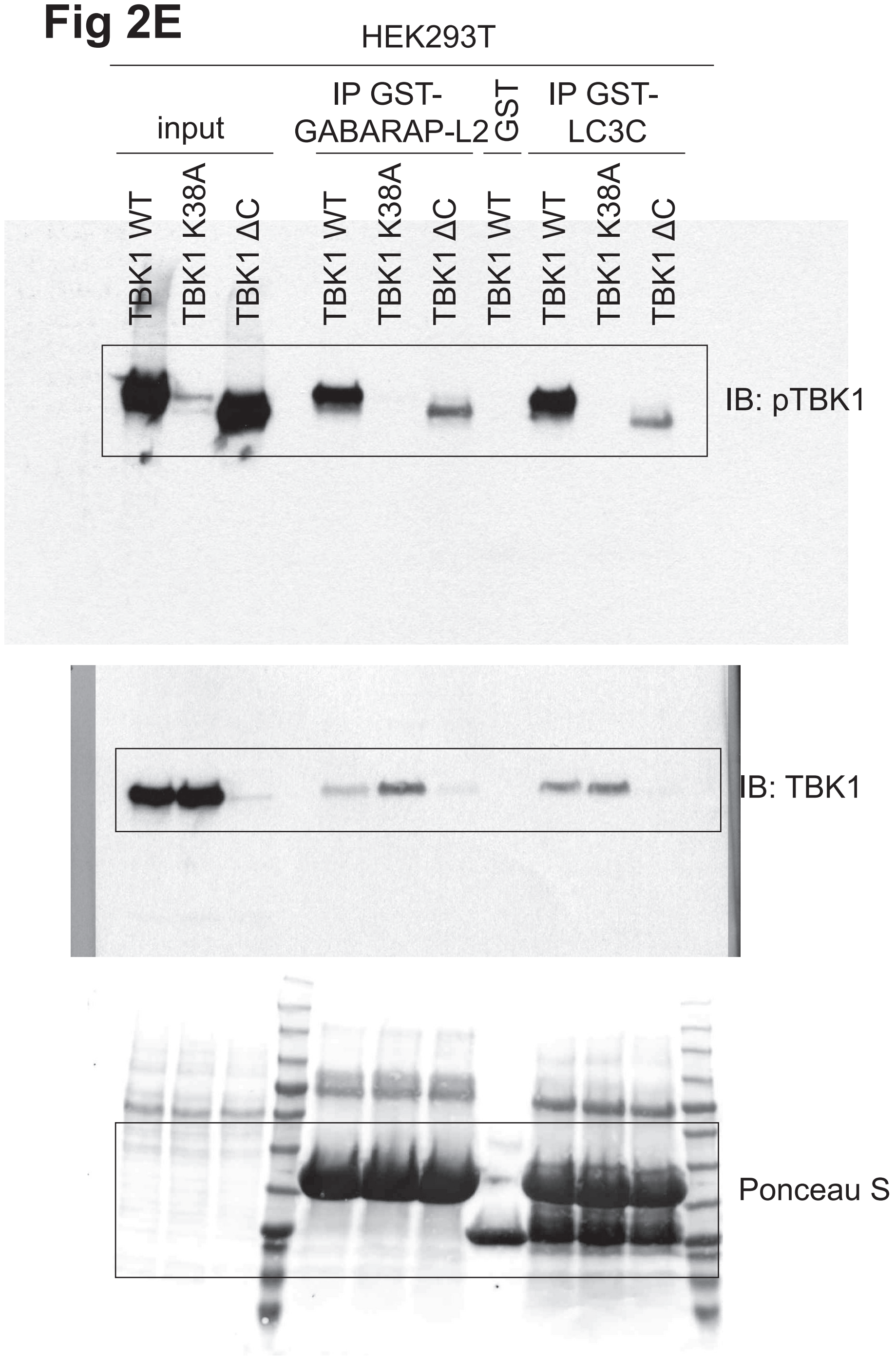
HEK293T
input
IP GST-GABARAP-L2
GST
IP GST-LC3C
TBK1 WT
TBK1 K38A
TBK1 ΔC
TBK1 WT
TBK1 K38A
TBK1 ΔC
TBK1 WT
TBK1 WT
TBK1 K38A
TBK1 ΔC
IB: pTBK1
IB: TBK1
Ponceau S
