# Supplementary material for: TBK1‐mediated phosphorylation of LC3C and GABARAP‐L2 controls autophagosome shedding by ATG4 protease
Source: EMBO Rep. 2019 Nov 11;21(1):e48317. doi: 10.15252/embr.201948317 (PMC6945063; doi:10.15252/embr.201948317)
Supplement: Supplementary file 15 — Source Data for Figure 8 [file EMBR-21-e48317-s013.pdf]

Fig 8A

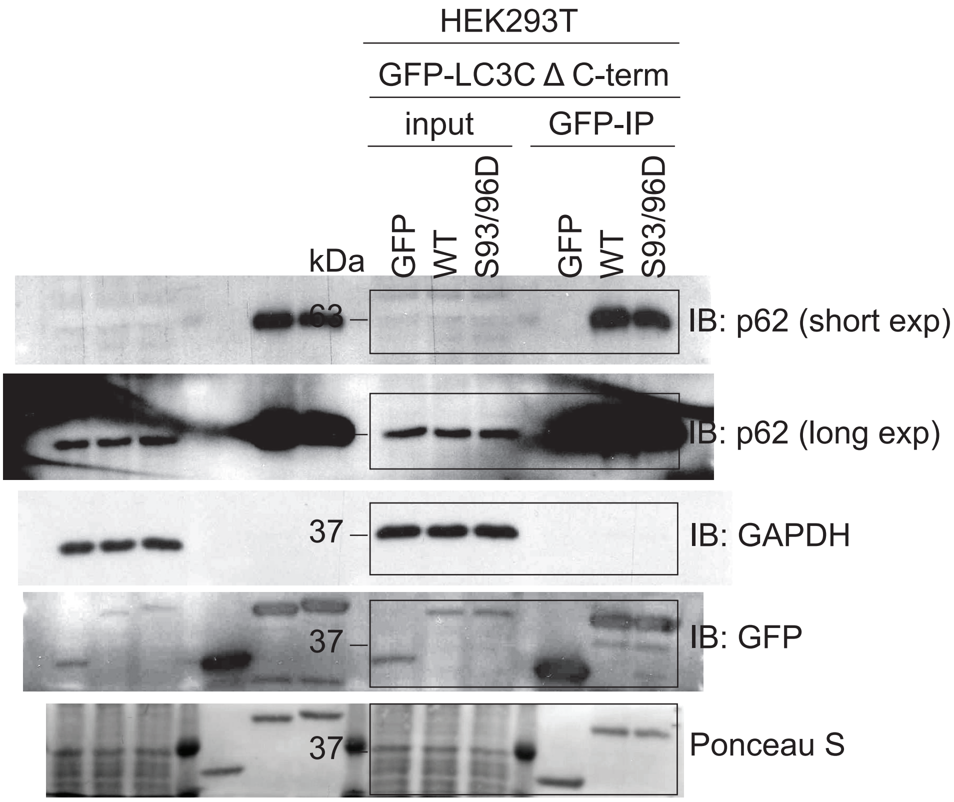

HEK293T
GFP-LC3C Δ C-term
input
GFP-IP
kDa
GFP
WT
S93/96D
GFP
WT
S93/96D
63
IB: p62 (short exp)
IB: p62 (long exp)
37
IB: GAPDH
37
IB: GFP
37
Ponceau S

Fig 8B

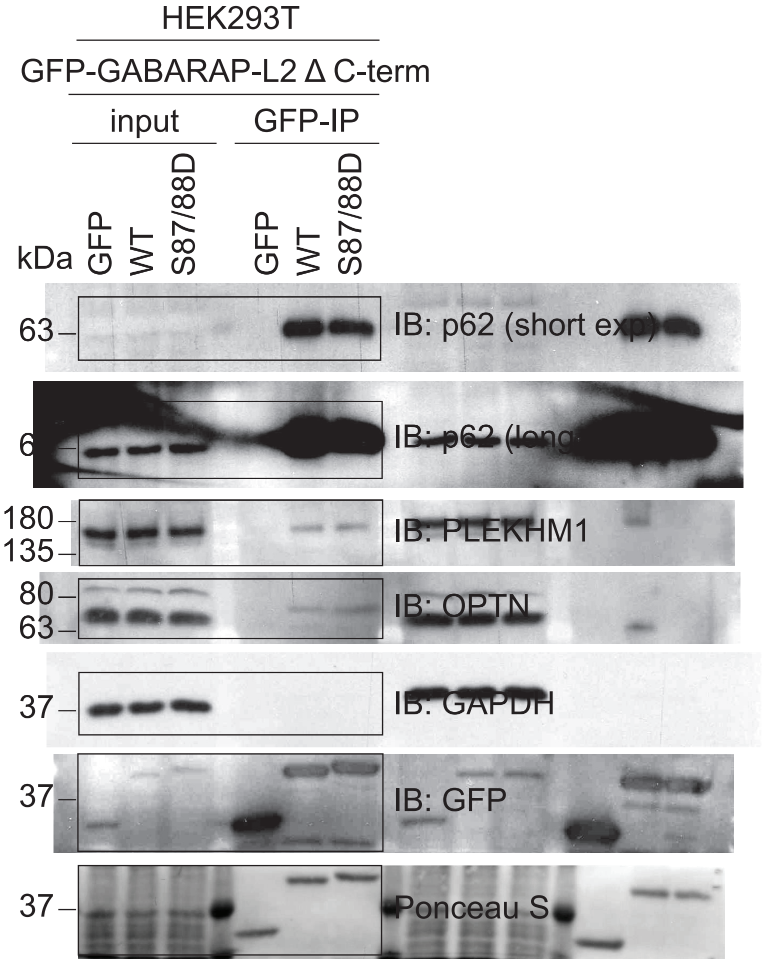

HEK293T
GFP-GABARAP-L2 Δ C-term
input
GFP-IP
kDa
GFP
WT
S87/88D
GFP
WT
S87/88D
63
IB: p62 (short exp)
180
135
IB: PLEKHM1
80
63
IB: OPTN
37
IB: GAPDH
37
IB: GFP
37
Ponceau S

Batch Analysis Report

Run Date: 8/8/19 1:39 PM Experiment: 190806_LinaH
User ID: GSHUser
Statistics Output: N/A
Worksheet PDF Output: C:\BDExport\Experiment\StefanS\190806_LinaH-Batch_Analysis_0808
2019133950.pdf

LC3C WT untreated

Tube	Status	Run Time
1	OK	8/8/19 1:39 PM
2	OK	8/8/19 1:39 PM
3	OK	8/8/19 1:39 PM
4	OK	8/8/19 1:39 PM
5	OK	8/8/19 1:40 PM
6	OK	8/8/19 1:40 PM

LC3C WT CCCP_16h

Tube	Status	Run Time
1	OK	8/8/19 1:40 PM
2	OK	8/8/19 1:40 PM
3	OK	8/8/19 1:40 PM
4	OK	8/8/19 1:40 PM
5	OK	8/8/19 1:40 PM
6	OK	8/8/19 1:40 PM

LC3C S9396A_ untreated

Tube	Status	Run Time
1	OK	8/8/19 1:40 PM
2	OK	8/8/19 1:40 PM
3	OK	8/8/19 1:40 PM
4	OK	8/8/19 1:40 PM
5	OK	8/8/19 1:40 PM
6	OK	8/8/19 1:40 PM

LC3C S9396A CCCP_16h

Tube	Status	Run Time
1	OK	8/8/19 1:40 PM
2	OK	8/8/19 1:40 PM

Tube	Status	Run Time
3	OK	8/8/19 1:40 PM
4	OK	8/8/19 1:40 PM
5	OK	8/8/19 1:40 PM
6	OK	8/8/19 1:40 PM

190806_LinaH

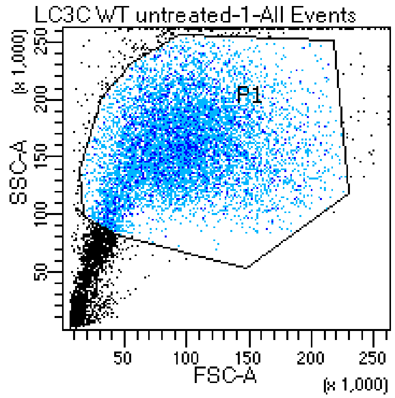

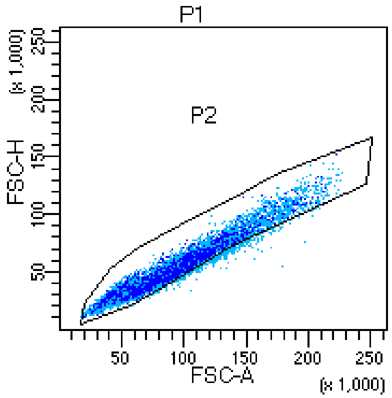

Experiment Name:	190806_LinaH
Specimen Name:	LC3C WT untreated
Tube Name:	1
Record Date:	Aug 6, 2019 10:00:57 AM

Population	#Events	%Parent	%Grand Pa...	mCherry T... Median	GFP FITC-A Median
All Events	11,729	####	####	28	121
P1	8,858	75.5	####	26	116
P2	8,274	93.4	70.5	26	115
mCherry pos	1,102	13.3	12.4	6,277	21,100
GFP pos	1,102	100.0	13.3	6,277	21,100

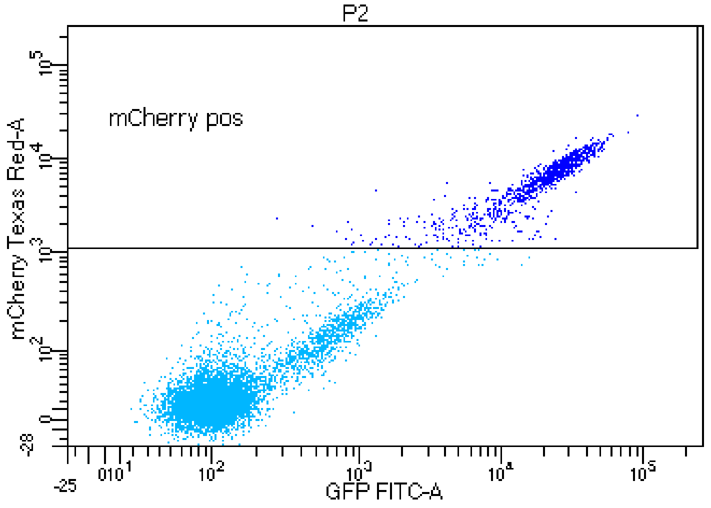

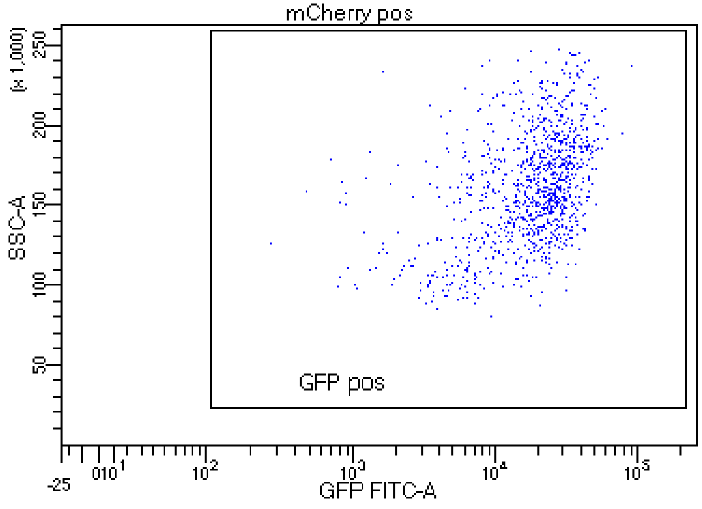

Tube: 1

Population	#Events	%Parent	%Total
All Events	11,729	####	100.0
P1	8,858	75.5	75.5
P2	8,274	93.4	70.5
mCherry pos	1,102	13.3	9.4
GFP pos	1,102	100.0	9.4

190806_LinaH

Experiment Name:	190806_LinaH
Specimen Name:	LC3C WT untreated
Tube Name:	2
Record Date:	Aug 6, 2019 10:03:26 AM

Population	#Events	%Parent	%Grand Pa...	mCherry T... Median	GFP FITC-A Median
All Events	12,010	####	####	29	119
P1	9,166	76.3	####	27	113
P2	8,582	93.6	71.5	27	112
mCherry pos	1,179	13.7	12.9	6,287	21,282
GFP pos	1,179	100.0	13.7	6,287	21,282

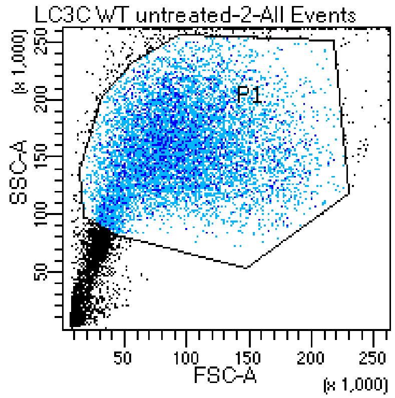

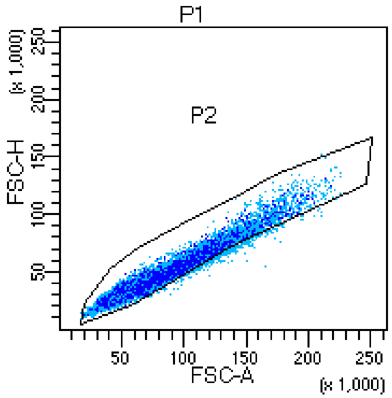

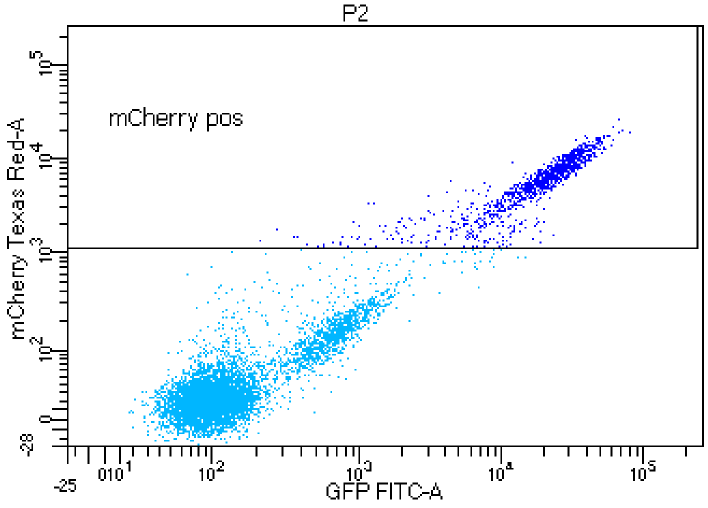

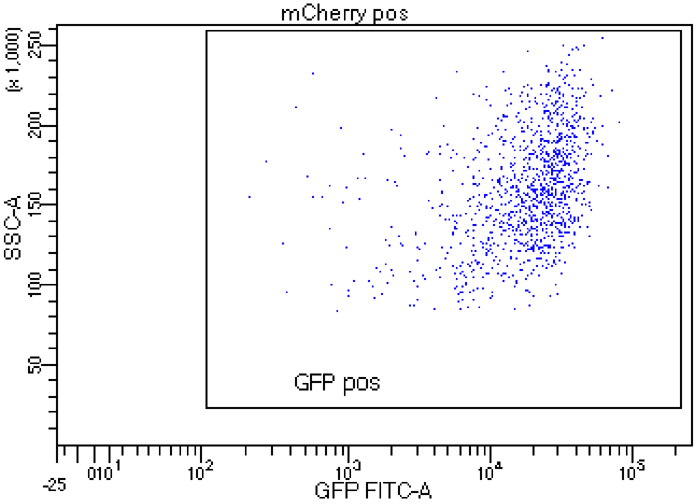

Tube: 2

Population	#Events	%Parent	%Total
All Events	12,010	####	100.0
P1	9,166	76.3	76.3
P2	8,582	93.6	71.5
mCherry pos	1,179	13.7	9.8
GFP pos	1,179	100.0	9.8

190806_LinaH

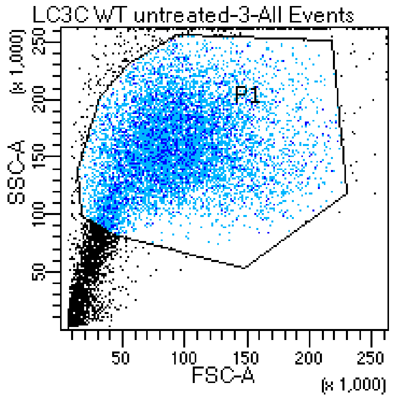

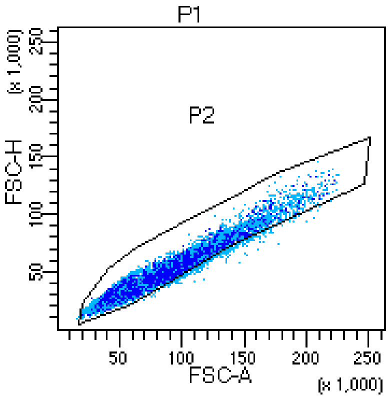

Experiment Name:	190806_LinaH
Specimen Name:	LC3C WT untreated
Tube Name:	3
Record Date:	Aug 6, 2019 10:04:30 AM

Population	#Events	%Parent	%Grand Pa...	mCherry T... Median	GFP FITC-A Median
All Events	12,253	####	####	30	127
P1	9,269	75.6	####	27	119
P2	8,715	94.0	71.1	27	117
mCherry pos	1,173	13.5	12.7	6,269	20,516
GFP pos	1,173	100.0	13.5	6,269	20,516

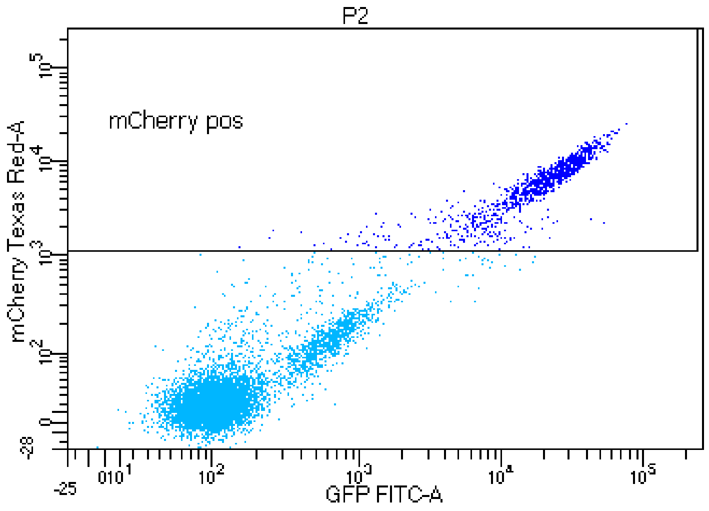

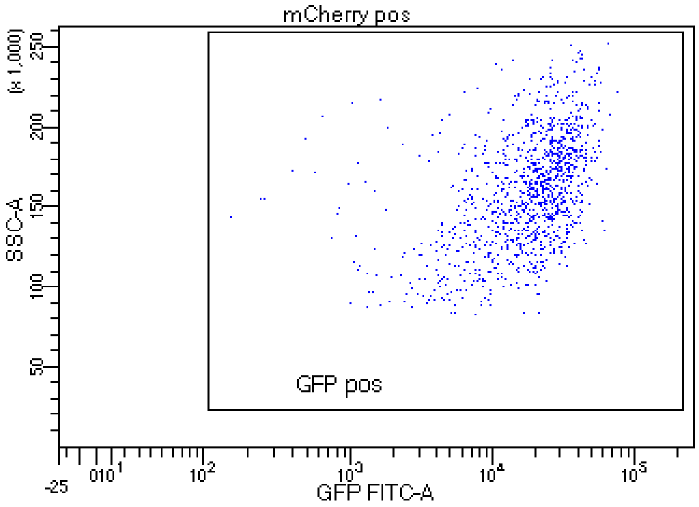

Tube: 3

Population	#Events	%Parent	%Total
All Events	12,253	####	100.0
P1	9,269	75.6	75.6
P2	8,715	94.0	71.1
mCherry pos	1,173	13.5	9.6
GFP pos	1,173	100.0	9.6

190806_LinaH

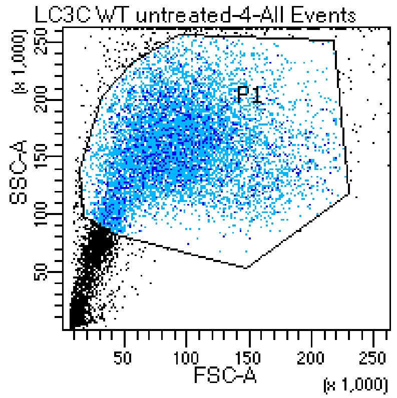

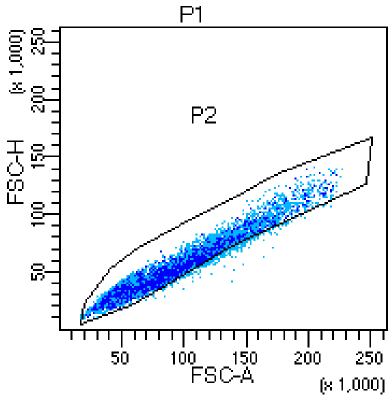

Experiment Name:	190806_LinaH
Specimen Name:	LC3C WT untreated
Tube Name:	4
Record Date:	Aug 6, 2019 10:05:30 AM

Population	#Events	%Parent	%Grand Pa...	mCherry T... Median	GFP FITC-A Median
All Events	12,019	####	####	29	119
P1	9,217	76.7	####	27	114
P2	8,656	93.9	72.0	26	112
mCherry pos	1,087	12.6	11.8	5,993	19,947
GFP pos	1,087	100.0	12.6	5,993	19,947

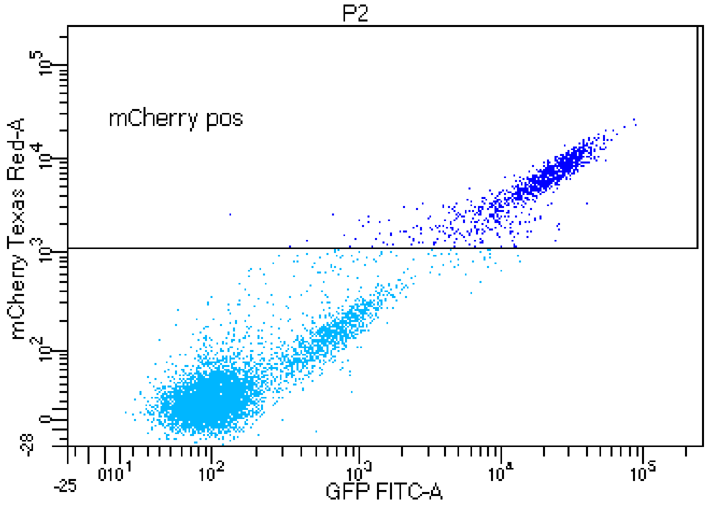

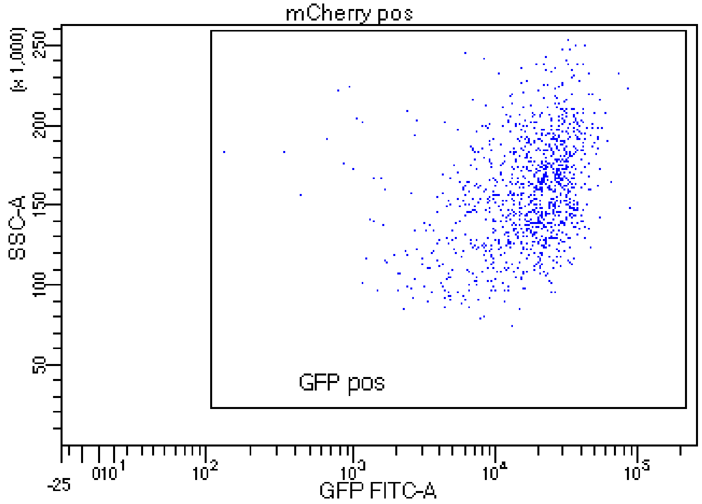

Tube: 4

Population	#Events	%Parent	%Total
All Events	12,019	####	100.0
P1	9,217	76.7	76.7
P2	8,656	93.9	72.0
mCherry pos	1,087	12.6	9.0
GFP pos	1,087	100.0	9.0

190806_LinaH

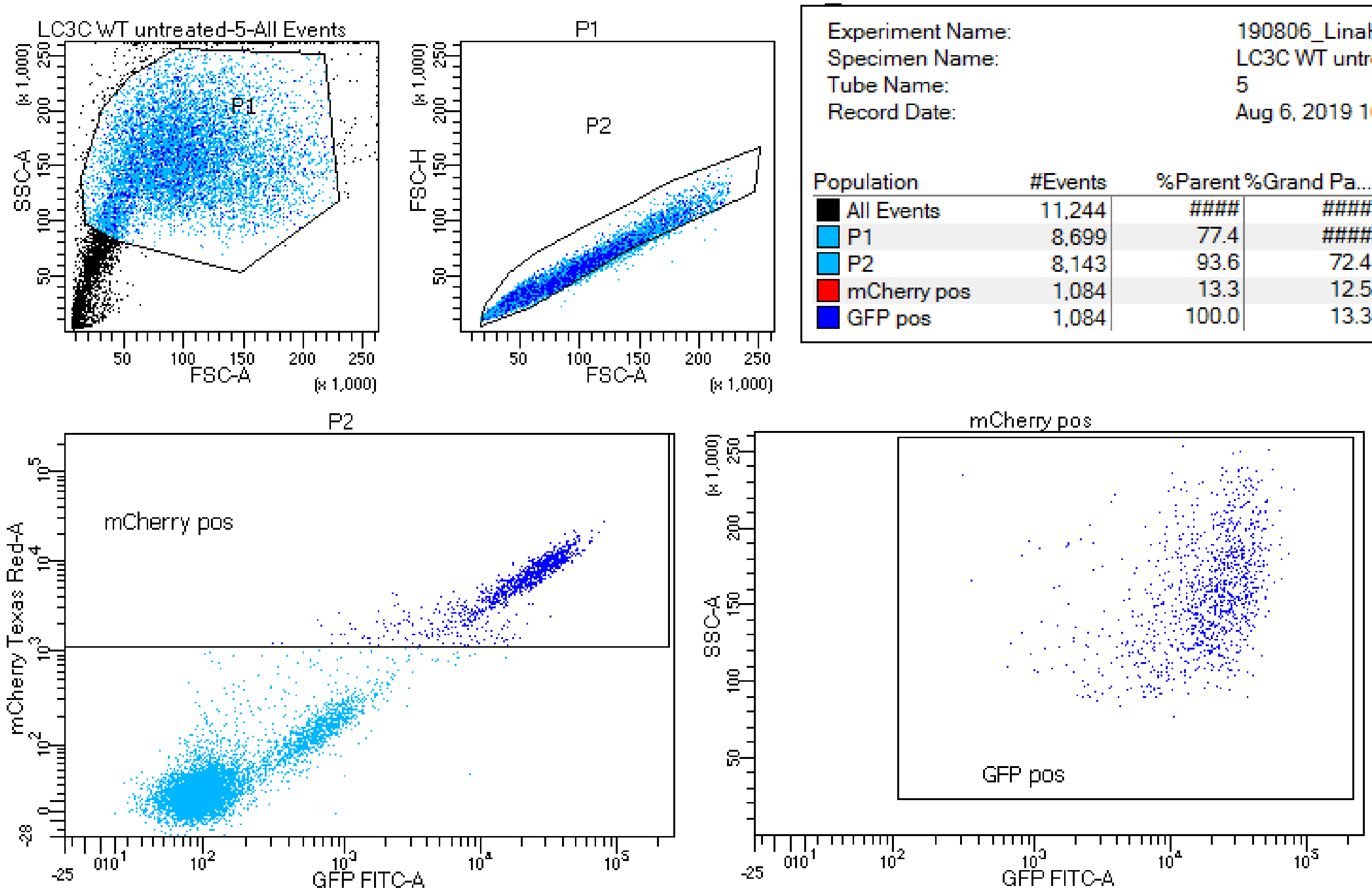

Experiment Name:	190806_LinaH
Specimen Name:	LC3C WT untreated
Tube Name:	5
Record Date:	Aug 6, 2019 10:06:27 AM

Population	#Events	%Parent	%Grand Pa...	mCherry T... Median	GFP FITC-A Median
All Events	11,244	####	####	30	115
P1	8,699	77.4	####	27	107
P2	8,143	93.6	72.4	27	105
mCherry pos	1,084	13.3	12.5	6,344	21,666
GFP pos	1,084	100.0	13.3	6,344	21,666

Tube: 5

Population	#Events	%Parent	%Total
All Events	11,244	####	100.0
P1	8,699	77.4	77.4
P2	8,143	93.6	72.4
mCherry pos	1,084	13.3	9.6
GFP pos	1,084	100.0	9.6

190806_LinaH

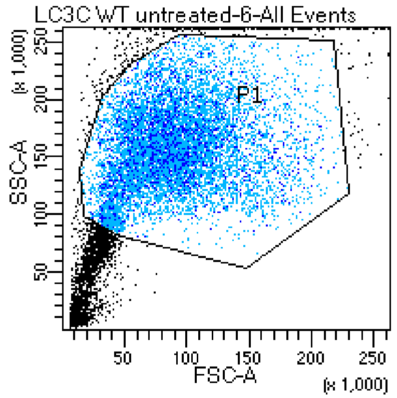

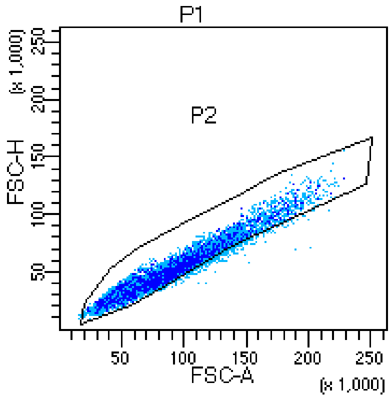

Experiment Name:	190806_LinaH
Specimen Name:	LC3C WT untreated
Tube Name:	6
Record Date:	Aug 6, 2019 10:07:25 AM

Population	#Events	%Parent	%Grand Pa...	mCherry T... Median	GFP FITC-A Median
All Events	12,193	####	####	29	119
P1	9,458	77.6	####	26	113
P2	8,906	94.2	73.0	26	112
mCherry pos	1,140	12.8	12.1	6,389	21,184
GFP pos	1,140	100.0	12.8	6,389	21,184

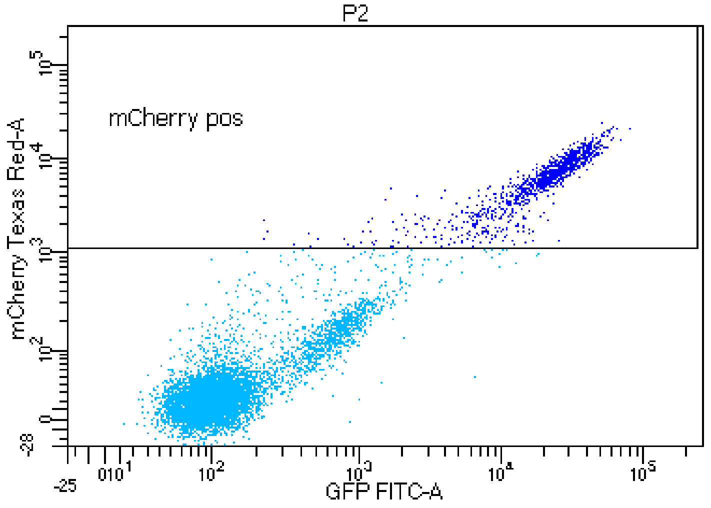

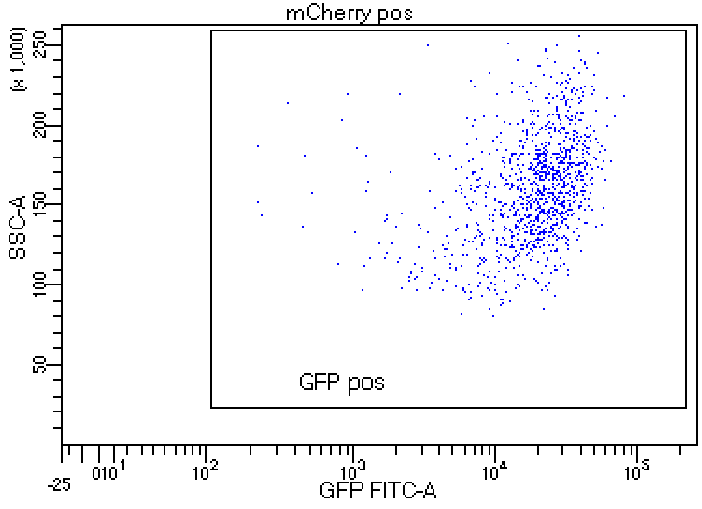

Tube: 6

Population	#Events	%Parent	%Total
All Events	12,193	####	100.0
P1	9,458	77.6	77.6
P2	8,906	94.2	73.0
mCherry pos	1,140	12.8	9.3
GFP pos	1,140	100.0	9.3

190806_LinaH

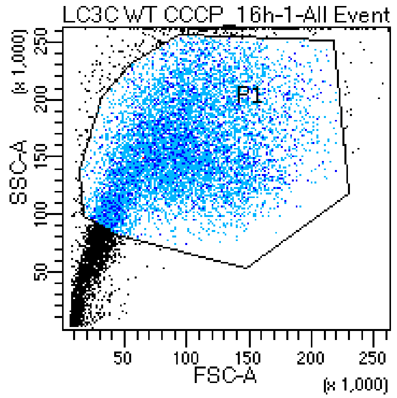

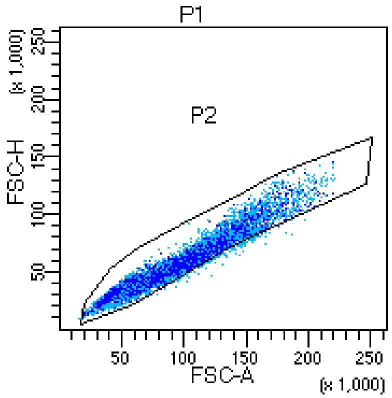

Experiment Name:	190806_LinaH
Specimen Name:	LC3C WT CCCP_16h
Tube Name:	1
Record Date:	Aug 6, 2019 10:09:33 AM

Population	#Events	%Parent	%Grand Pa...	mCherry T... Median	GFP FITC-A Median
All Events	12,333	####	####	46	133
P1	9,163	74.3	####	41	116
P2	8,867	96.8	71.9	41	115
mCherry pos	1,174	13.2	12.8	2,844	6,259
GFP pos	1,173	99.9	13.2	2,846	6,260

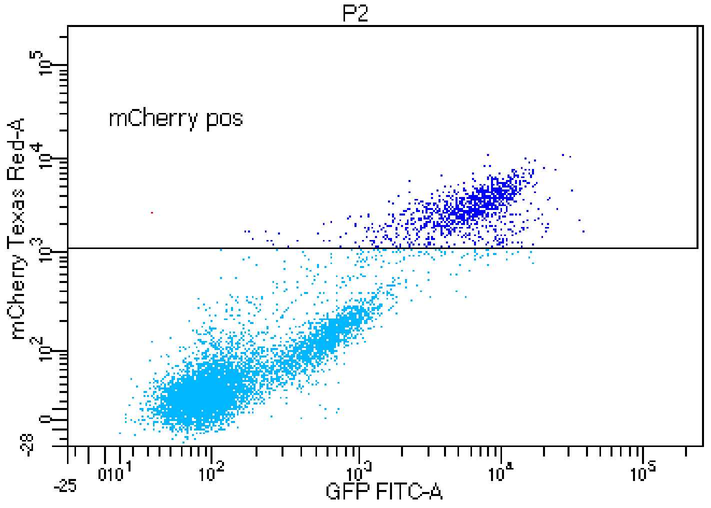

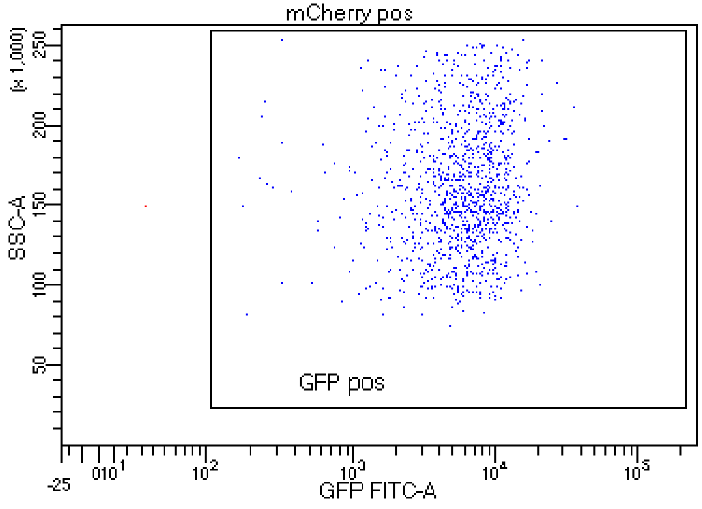

Tube: 1

Population	#Events	%Parent	%Total
All Events	12,333	####	100.0
P1	9,163	74.3	74.3
P2	8,867	96.8	71.9
mCherry pos	1,174	13.2	9.5
GFP pos	1,173	99.9	9.5

190806_LinaH

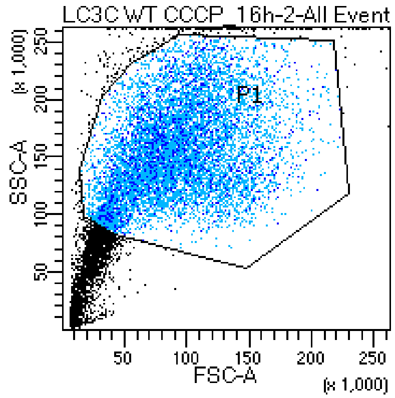

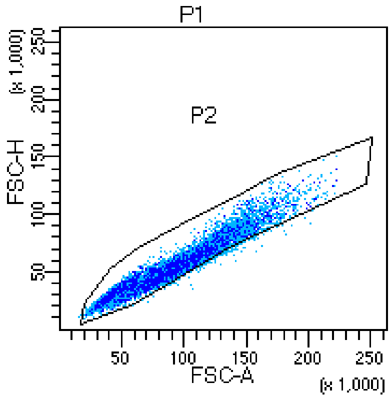

Experiment Name: 190806_LinaH
Specimen Name: LC3C WT CCCP_16h
Tube Name: 2
Record Date: Aug 6, 2019 10:10:58 AM

Population	#Events	%Parent	%Grand Pa...	mCherry T... Median	GFP FITC-A Median
All Events	12,050	####	####	42	127
P1	9,066	75.2	####	39	115
P2	8,758	96.6	72.7	39	114
mCherry pos	1,171	13.4	12.9	2,939	5,004
GFP pos	1,171	100.0	13.4	2,939	5,004

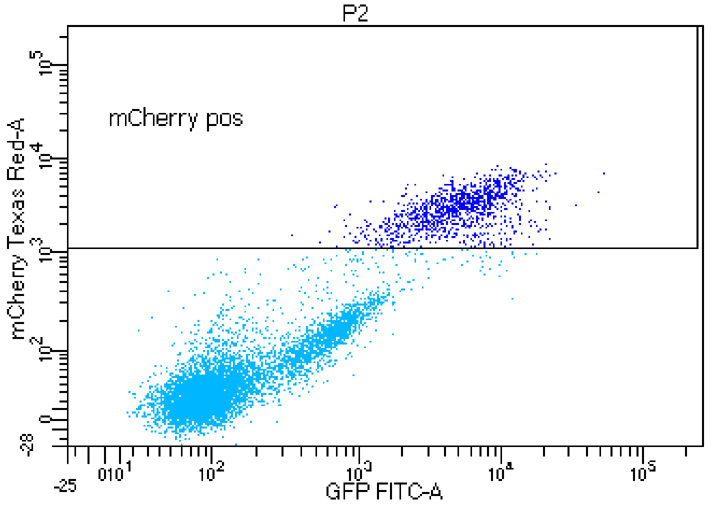

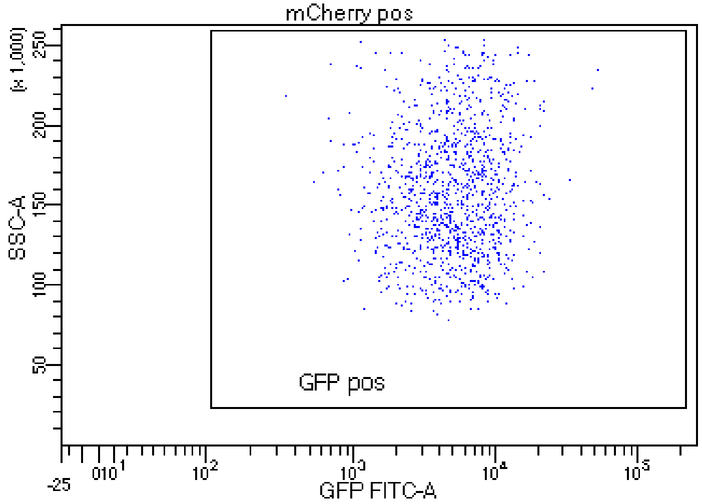

Tube: 2

Population	#Events	%Parent	%Total
All Events	12,050	####	100.0
P1	9,066	75.2	75.2
P2	8,758	96.6	72.7
mCherry pos	1,171	13.4	9.7
GFP pos	1,171	100.0	9.7

190806_LinaH

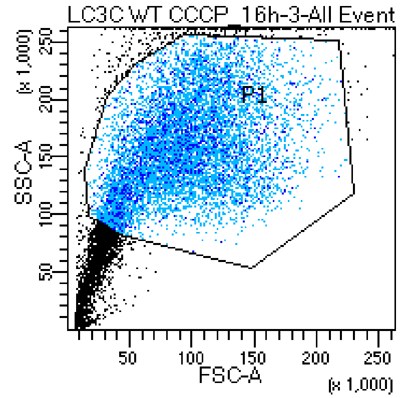

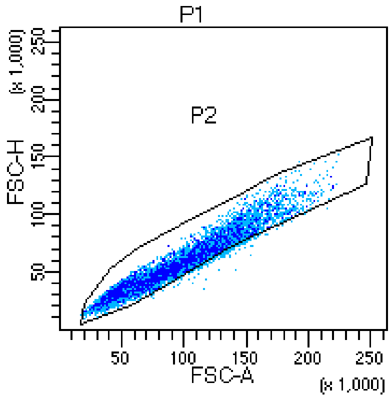

Experiment Name:	190806_LinaH
Specimen Name:	LC3C WT CCCP_16h
Tube Name:	3
Record Date:	Aug 6, 2019 10:12:20 AM

Population	#Events	%Parent	%Grand Pa...	mCherry T... Median	GFP FITC-A Median
All Events	12,617	####	####	45	130
P1	9,459	75.0	####	40	112
P2	9,169	96.9	72.7	39	111
mCherry pos	1,175	12.8	12.4	2,993	6,330
GFP pos	1,175	100.0	12.8	2,993	6,330

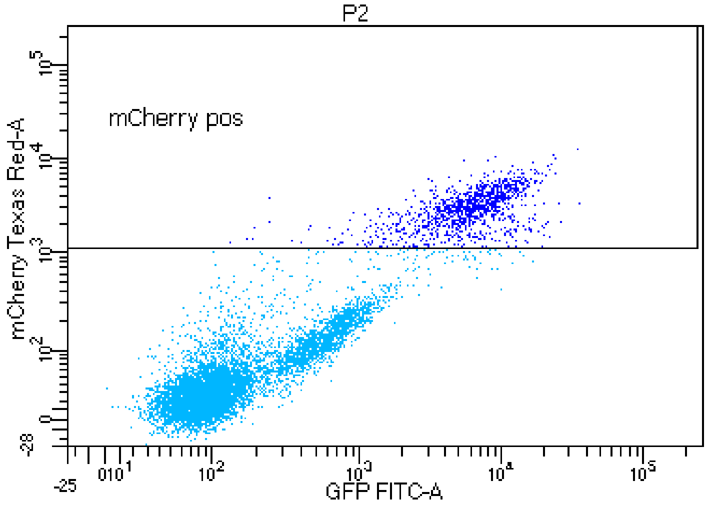

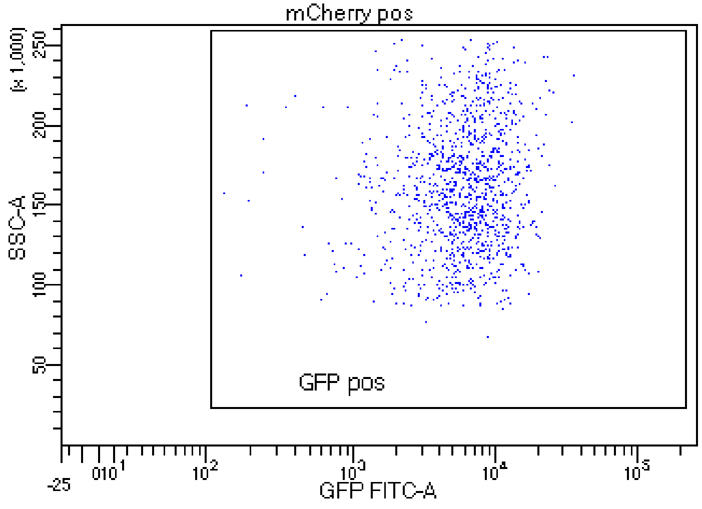

Tube: 3

Population	#Events	%Parent	%Total
All Events	12,617	####	100.0
P1	9,459	75.0	75.0
P2	9,169	96.9	72.7
mCherry pos	1,175	12.8	9.3
GFP pos	1,175	100.0	9.3

190806_LinaH

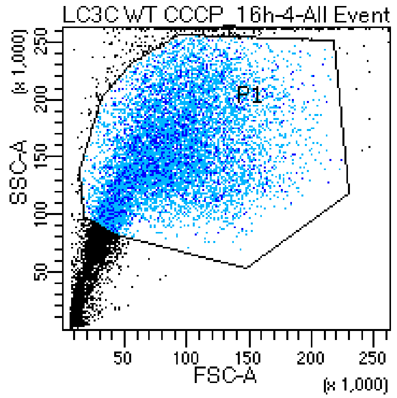

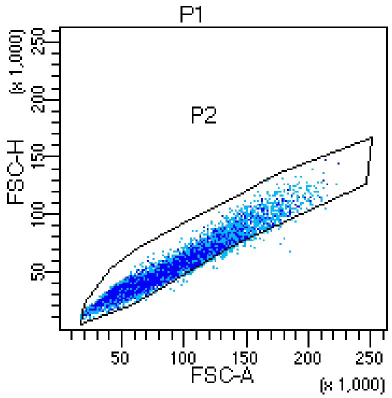

Experiment Name: 190806_LinaH
Specimen Name: LC3C WT CCCP_16h
Tube Name: 4
Record Date: Aug 6, 2019 10:13:43 AM

Population	#Events	%Parent	%Grand Pa...	mCherry T... Median	GFP FITC-A Median
All Events	13,391	####	####	44	129
P1	9,936	74.2	####	38	113
P2	9,644	97.1	72.0	38	111
mCherry pos	1,292	13.4	13.0	2,940	5,375
GFP pos	1,292	100.0	13.4	2,940	5,375

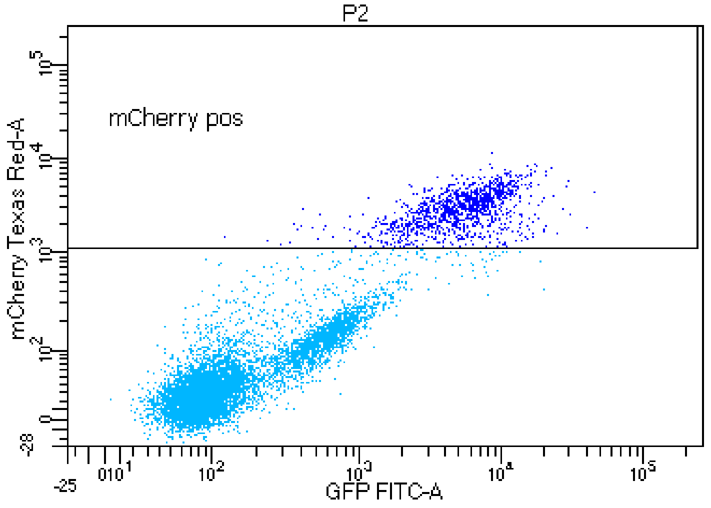

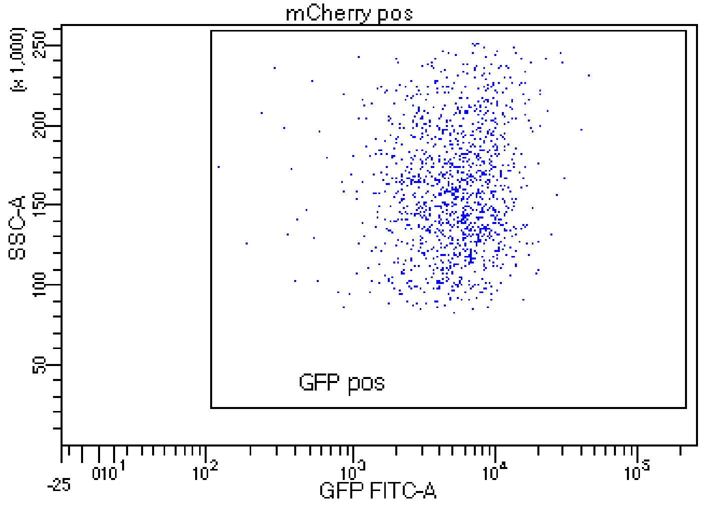

Tube: 4

Population	#Events	%Parent	%Total
All Events	13,391	####	100.0
P1	9,936	74.2	74.2
P2	9,644	97.1	72.0
mCherry pos	1,292	13.4	9.6
GFP pos	1,292	100.0	9.6

190806_LinaH

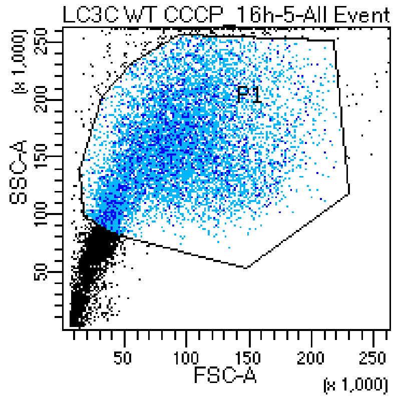

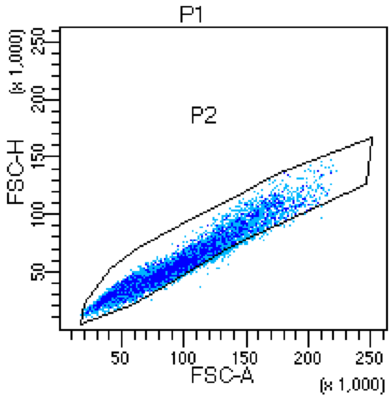

Experiment Name:	190806_LinaH
Specimen Name:	LC3C WT CCCP_16h
Tube Name:	5
Record Date:	Aug 6, 2019 10:14:51 AM

Population	#Events	%Parent	%Grand Pa...	mCherry T... Median	GFP FITC-A Median
All Events	13,566	####	####	47	135
P1	9,792	72.2	####	42	117
P2	9,481	96.8	69.9	42	115
mCherry pos	1,296	13.7	13.2	2,839	6,039
GFP pos	1,296	100.0	13.7	2,839	6,039

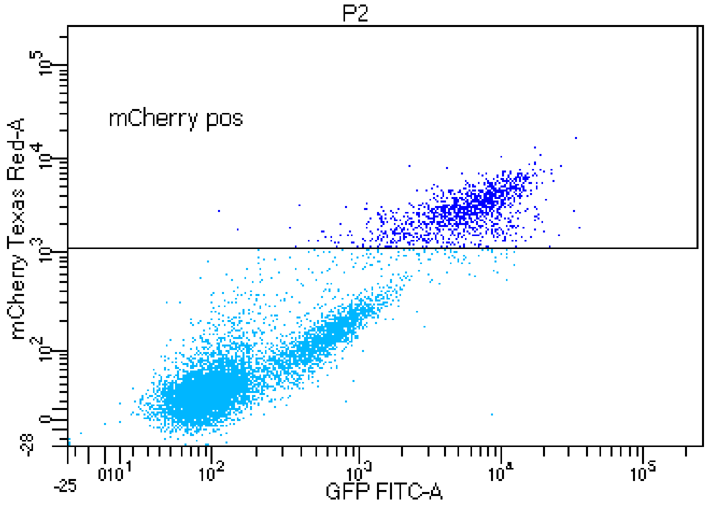

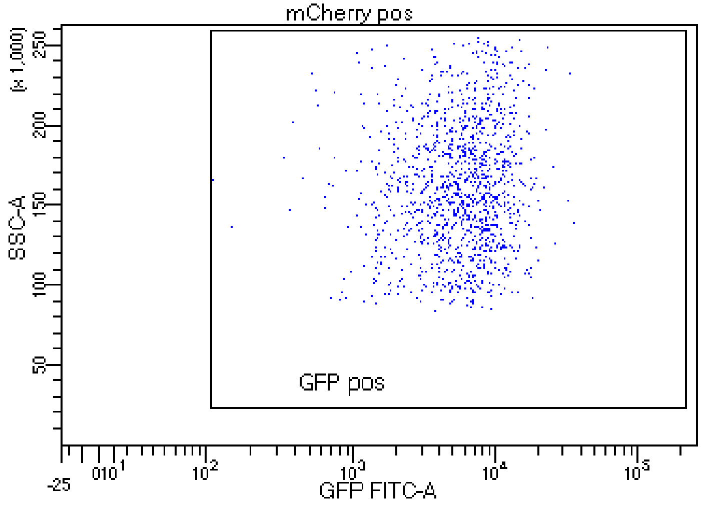

Tube: 5

Population	#Events	%Parent	%Total
All Events	13,566	####	100.0
P1	9,792	72.2	72.2
P2	9,481	96.8	69.9
mCherry pos	1,296	13.7	9.6
GFP pos	1,296	100.0	9.6

190806_LinaH

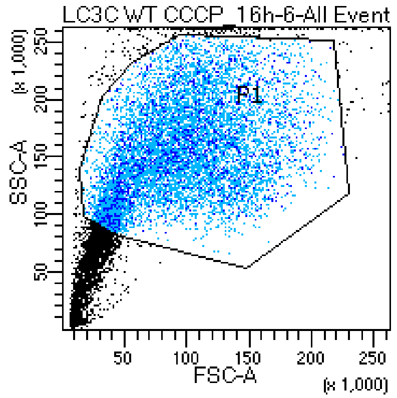

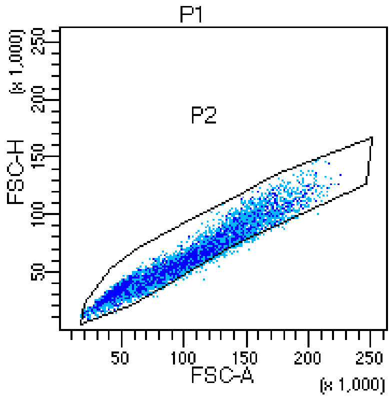

Experiment Name:	190806_LinaH
Specimen Name:	LC3C WT CCCP_16h
Tube Name:	6
Record Date:	Aug 6, 2019 10:16:00 AM

Population	#Events	%Parent	%Grand Pa...	mCherry T... Median	GFP FITC-A Median
All Events	12,731	####	####	49	146
P1	9,188	72.2	####	44	121
P2	8,871	96.5	69.7	43	119
mCherry pos	1,198	13.5	13.0	2,883	6,017
GFP pos	1,198	100.0	13.5	2,883	6,017

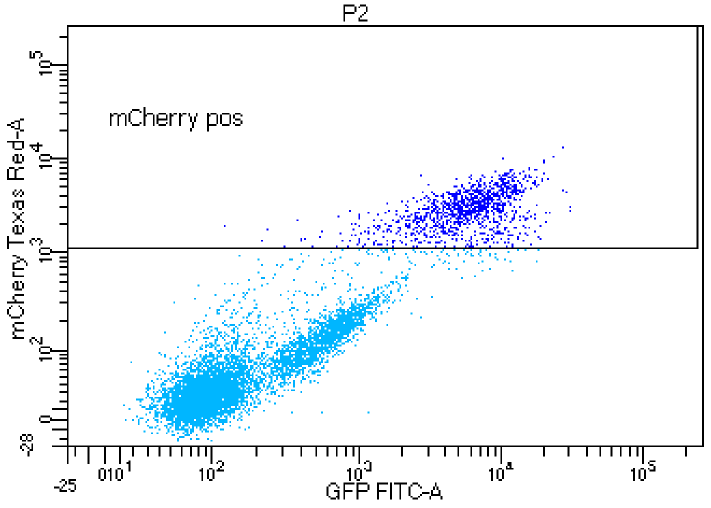

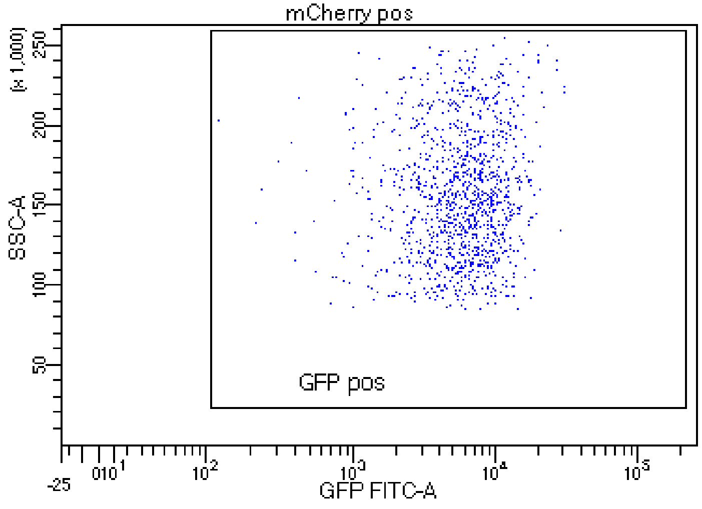

Tube: 6

Population	#Events	%Parent	%Total
All Events	12,731	####	100.0
P1	9,188	72.2	72.2
P2	8,871	96.5	69.7
mCherry pos	1,198	13.5	9.4
GFP pos	1,198	100.0	9.4

190806_LinaH

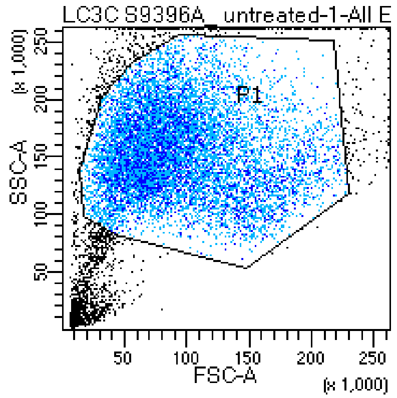

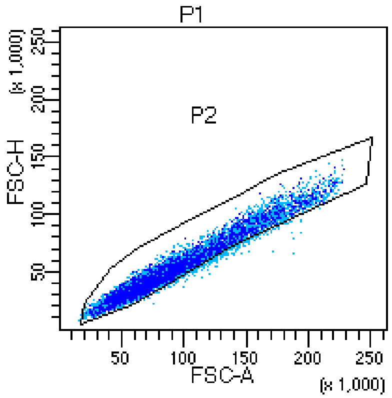

Experiment Name:	190806_LinaH
Specimen Name:	LC3C S9396A_ untreated
Tube Name:	1
Record Date:	Aug 6, 2019 10:17:46 AM

Population	#Events	%Parent	%Grand Pa...	mCherry T... Median	GFP FITC-A Median
All Events	12,276	####	####	22	97
P1	10,339	84.2	####	22	95
P2	9,824	95.0	80.0	21	94
mCherry pos	1,913	19.5	18.5	5,255	18,115
GFP pos	1,913	100.0	19.5	5,255	18,115

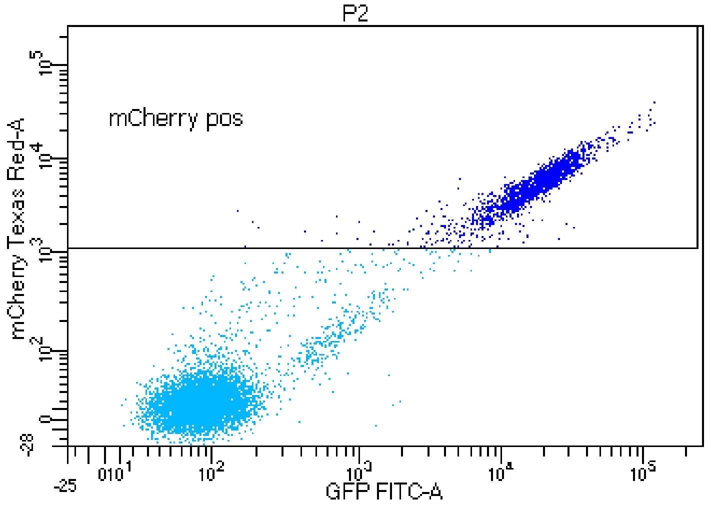

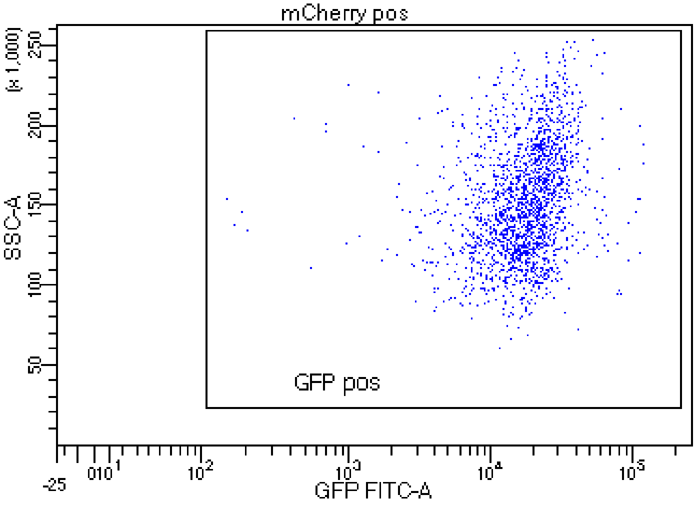

Tube: 1

Population	#Events	%Parent	%Total
All Events	12,276	####	100.0
P1	10,339	84.2	84.2
P2	9,824	95.0	80.0
mCherry pos	1,913	19.5	15.6
GFP pos	1,913	100.0	15.6

190806_LinaH

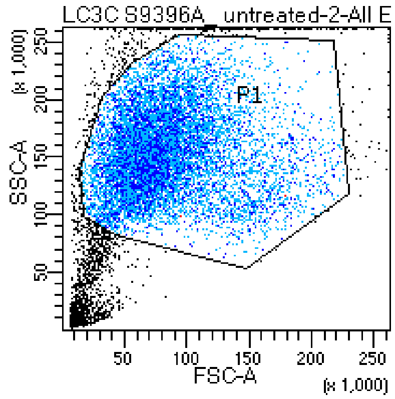

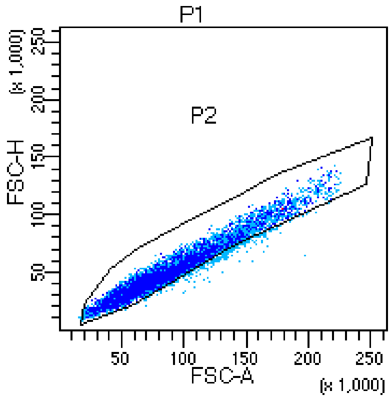

Experiment Name:	190806_LinaH
Specimen Name:	LC3C S9396A_ untreated
Tube Name:	2
Record Date:	Aug 6, 2019 10:18:38 AM

Population	#Events	%Parent	%Grand Pa...	mCherry T... Median	GFP FITC-A Median
All Events	13,052	####	####	23	108
P1	10,977	84.1	####	23	108
P2	10,482	95.5	80.3	22	106
mCherry pos	2,075	19.8	18.9	5,517	18,268
GFP pos	2,072	99.9	19.8	5,525	18,303

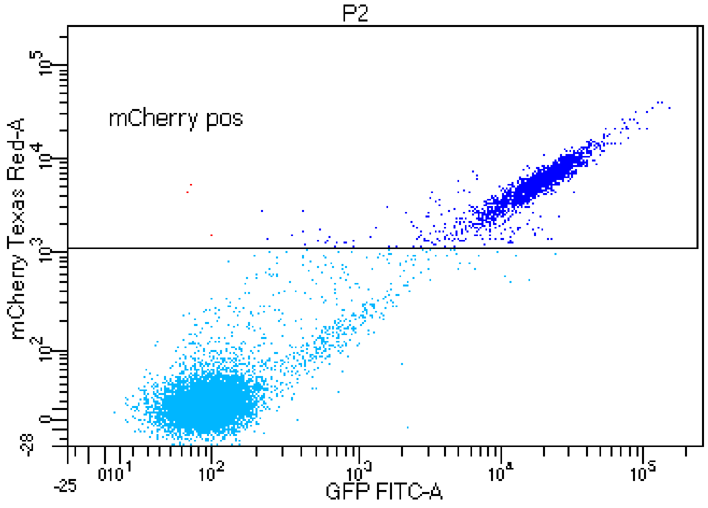

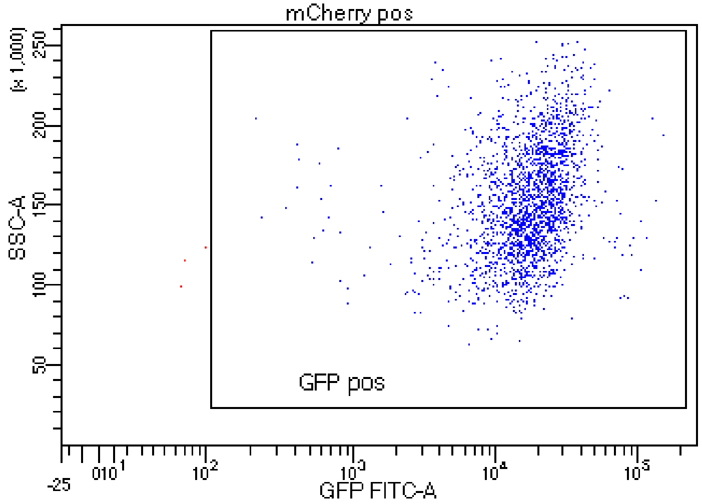

Tube: 2

Population	#Events	%Parent	%Total
All Events	13,052	####	100.0
P1	10,977	84.1	84.1
P2	10,482	95.5	80.3
mCherry pos	2,075	19.8	15.9
GFP pos	2,072	99.9	15.9

190806_LinaH

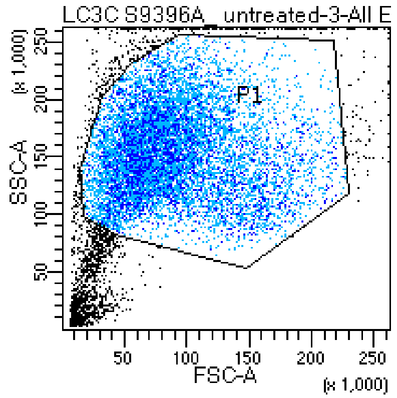

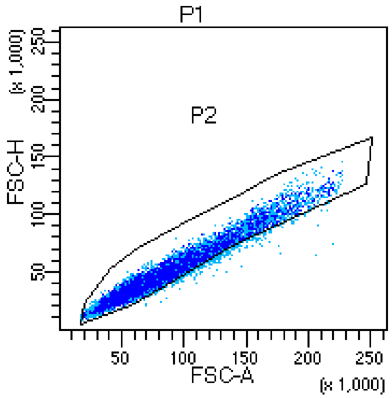

Experiment Name:	190806_LinaH
Specimen Name:	LC3C S9396A_ untreated
Tube Name:	3
Record Date:	Aug 6, 2019 10:19:38 AM

Population	#Events	%Parent	%Grand Pa...	mCherry T... Median	GFP FITC-A Median
All Events	12,486	####	####	23	99
P1	10,573	84.7	####	23	97
P2	10,032	94.9	80.3	23	96
mCherry pos	1,969	19.6	18.6	5,667	18,639
GFP pos	1,969	100.0	19.6	5,667	18,639

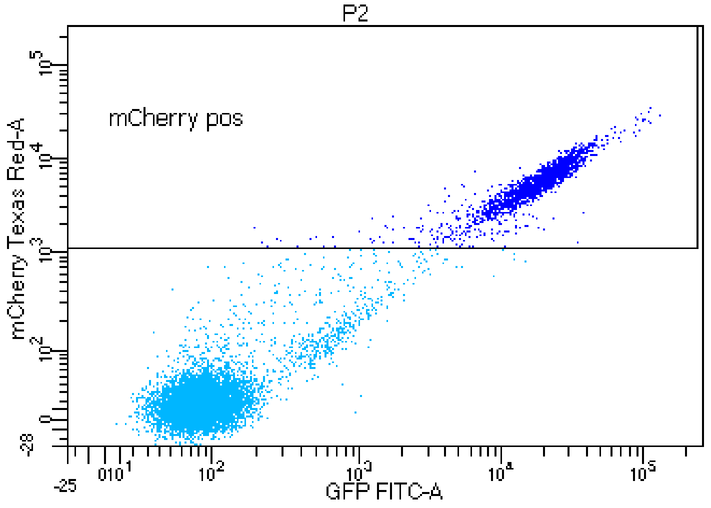

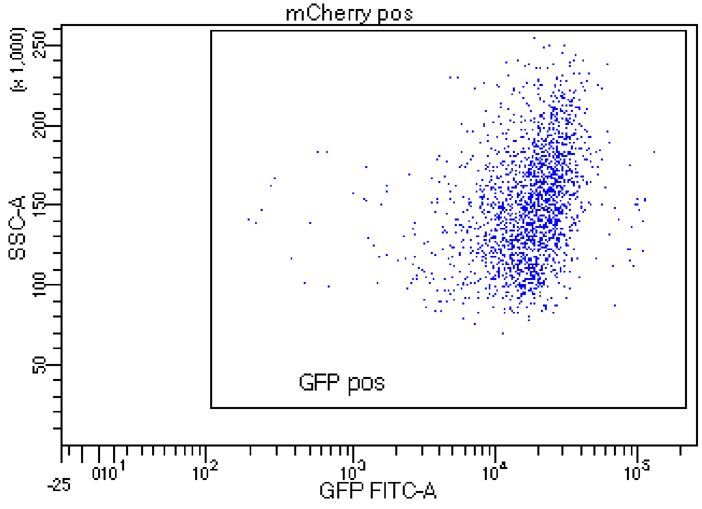

Tube: 3

Population	#Events	%Parent	%Total
All Events	12,486	####	100.0
P1	10,573	84.7	84.7
P2	10,032	94.9	80.3
mCherry pos	1,969	19.6	15.8
GFP pos	1,969	100.0	15.8

190806_LinaH

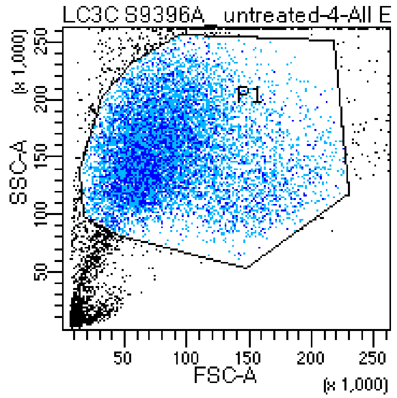

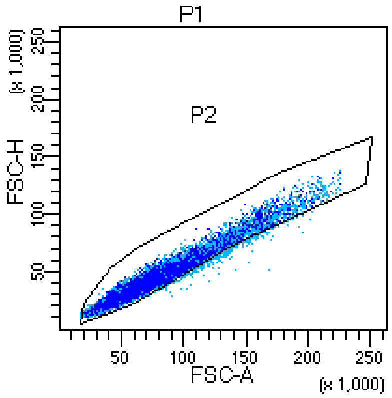

Experiment Name:	190806_LinaH
Specimen Name:	LC3C S9396A_ untreated
Tube Name:	4
Record Date:	Aug 6, 2019 10:20:46 AM

Population	#Events	%Parent	%Grand Pa...	mCherry T... Median	GFP FITC-A Median
All Events	12,922	####	####	23	104
P1	10,924	84.5	####	23	102
P2	10,432	95.5	80.7	23	101
mCherry pos	2,113	20.3	19.3	5,454	18,093
GFP pos	2,113	100.0	20.3	5,454	18,093

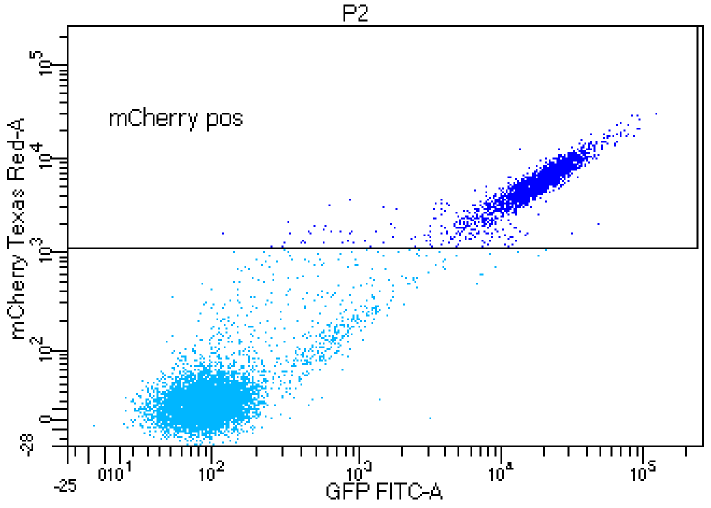

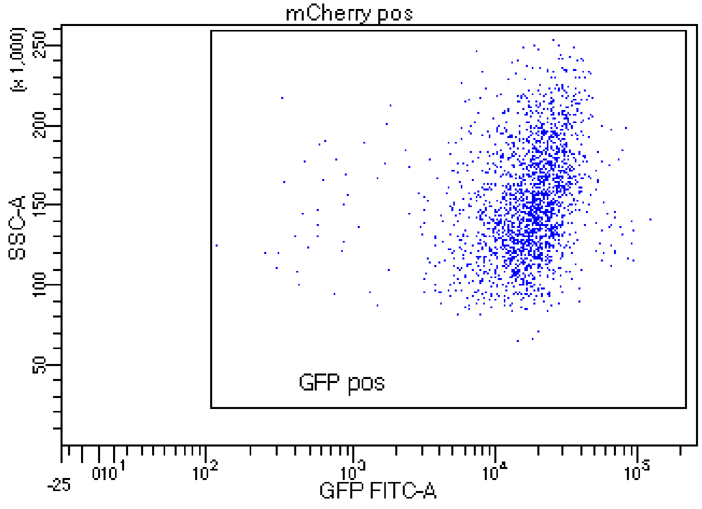

Tube: 4

Population	#Events	%Parent	%Total
All Events	12,922	####	100.0
P1	10,924	84.5	84.5
P2	10,432	95.5	80.7
mCherry pos	2,113	20.3	16.4
GFP pos	2,113	100.0	16.4

190806_LinaH

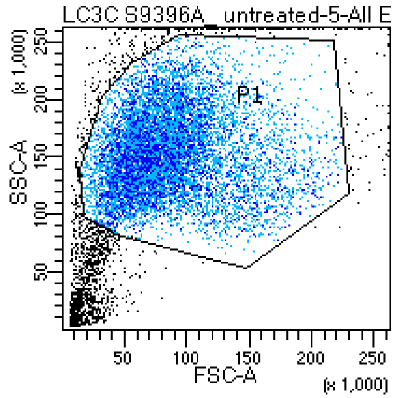

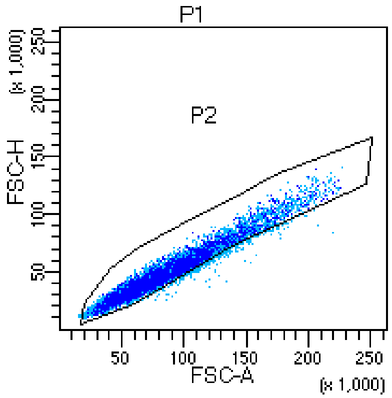

Experiment Name:	190806_LinaH
Specimen Name:	LC3C S9396A_ untreated
Tube Name:	5
Record Date:	Aug 6, 2019 10:21:49 AM

Population	#Events	%Parent	%Grand Pa...	mCherry T... Median	GFP FITC-A Median
All Events	12,386	####	####	22	99
P1	10,531	85.0	####	22	98
P2	10,047	95.4	81.1	21	97
mCherry pos	2,018	20.1	19.2	5,459	18,294
GFP pos	2,017	100.0	20.1	5,459	18,295

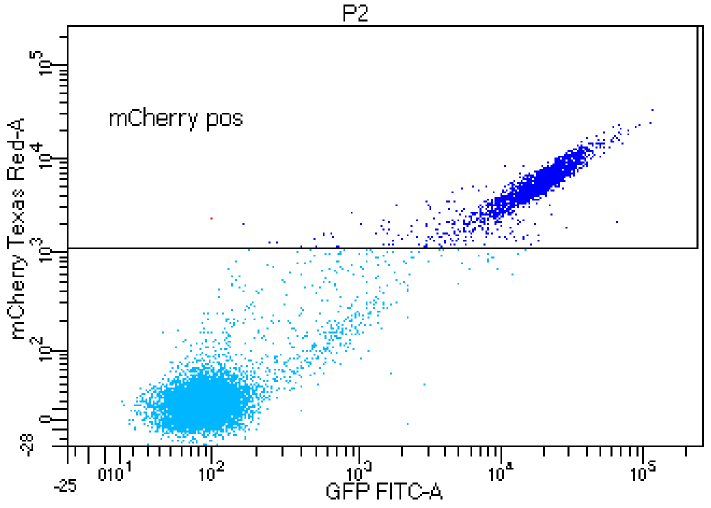

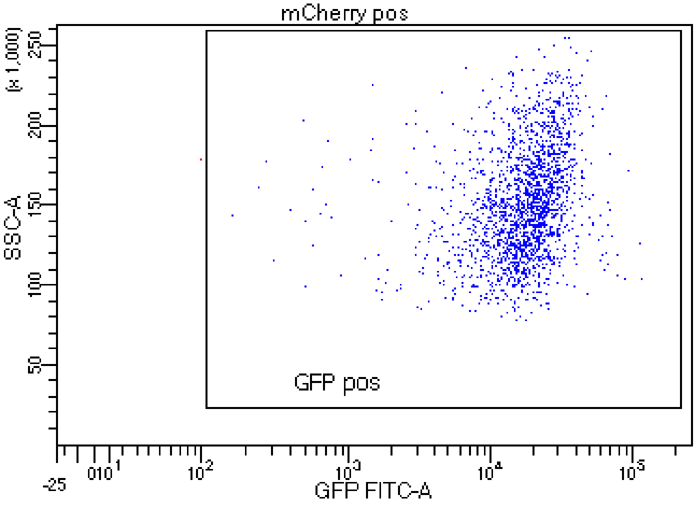

Tube: 5

Population	#Events	%Parent	%Total
All Events	12,386	####	100.0
P1	10,531	85.0	85.0
P2	10,047	95.4	81.1
mCherry pos	2,018	20.1	16.3
GFP pos	2,017	100.0	16.3

190806_LinaH

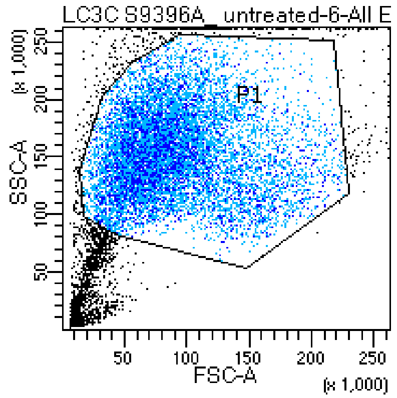

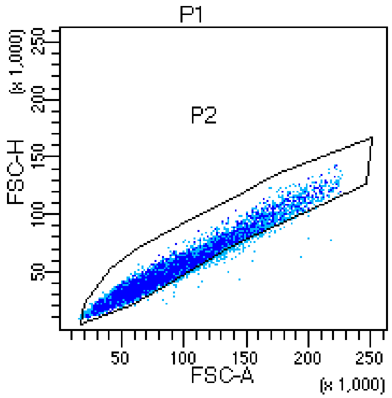

Experiment Name:	190806_LinaH
Specimen Name:	LC3C S9396A_ untreated
Tube Name:	6
Record Date:	Aug 6, 2019 10:22:49 AM

Population	#Events	%Parent	%Grand Pa...	mCherry T... Median	GFP FITC-A Median
All Events	12,395	####	####	24	104
P1	10,445	84.3	####	23	101
P2	9,890	94.7	79.8	23	101
mCherry pos	1,960	19.8	18.8	5,532	18,050
GFP pos	1,960	100.0	19.8	5,532	18,050

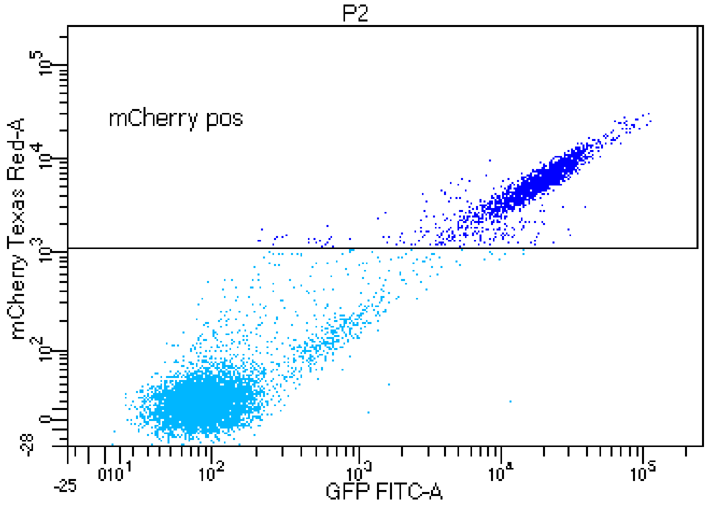

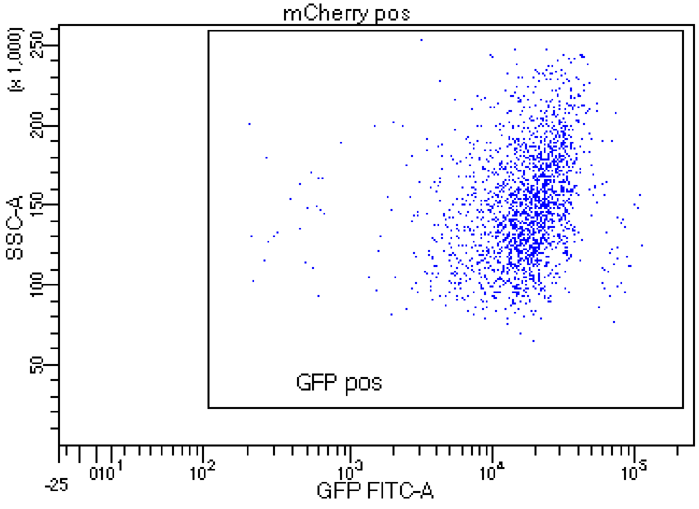

Tube: 6

Population	#Events	%Parent	%Total
All Events	12,395	####	100.0
P1	10,445	84.3	84.3
P2	9,890	94.7	79.8
mCherry pos	1,960	19.8	15.8
GFP pos	1,960	100.0	15.8

190806_LinaH

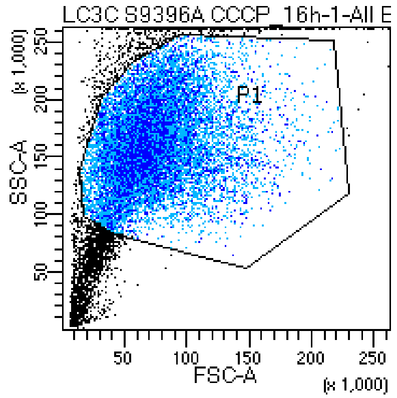

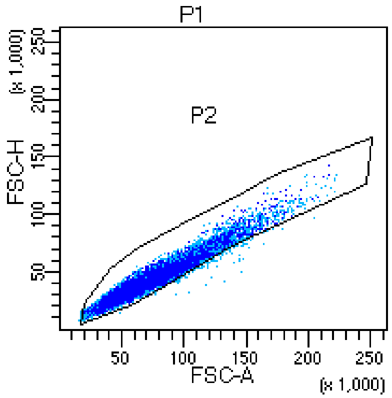

Experiment Name:	190806_LinaH
Specimen Name:	LC3C S9396A CCCP_16h
Tube Name:	1
Record Date:	Aug 6, 2019 10:25:03 AM

Population	#Events	%Parent	%Grand Pa...	mCherry T... Median	GFP FITC-A Median
All Events	18,450	####	####	31	89
P1	15,547	84.3	####	29	85
P2	15,293	98.4	82.9	29	84
mCherry pos	2,937	19.2	18.9	2,534	4,206
GFP pos	2,937	100.0	19.2	2,534	4,206

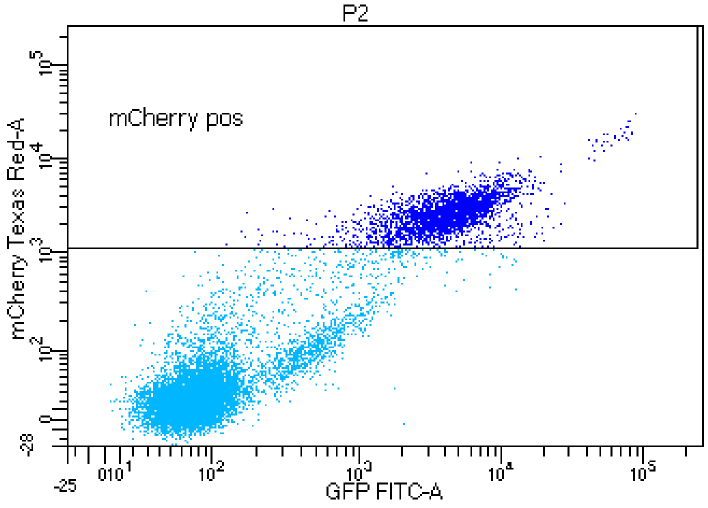

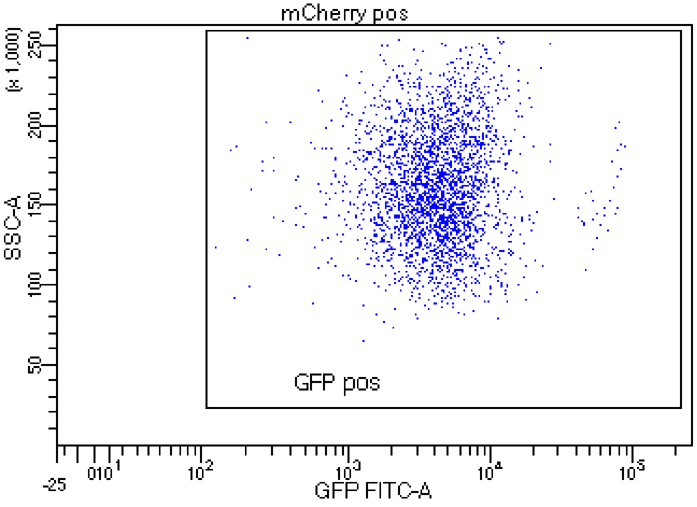

Tube: 1

Population	#Events	%Parent	%Total
All Events	18,450	####	100.0
P1	15,547	84.3	84.3
P2	15,293	98.4	82.9
mCherry pos	2,937	19.2	15.9
GFP pos	2,937	100.0	15.9

190806_LinaH

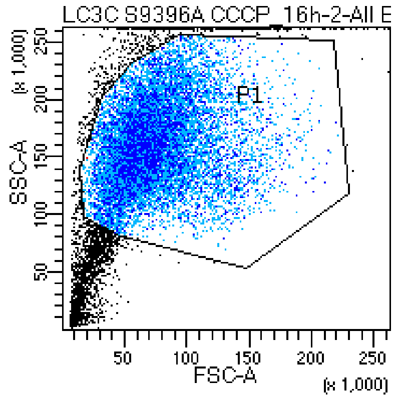

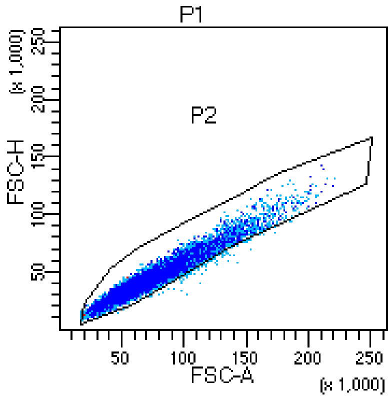

Experiment Name:	190806_LinaH
Specimen Name:	LC3C S9396A CCCP_16h
Tube Name:	2
Record Date:	Aug 6, 2019 10:26:19 AM

Population	#Events	%Parent	%Grand Pa...	mCherry T... Median	GFP FITC-A Median
All Events	17,151	####	####	32	88
P1	14,578	85.0	####	31	85
P2	14,346	98.4	83.6	30	84
mCherry pos	2,791	19.5	19.1	2,541	4,216
GFP pos	2,789	99.9	19.4	2,542	4,216

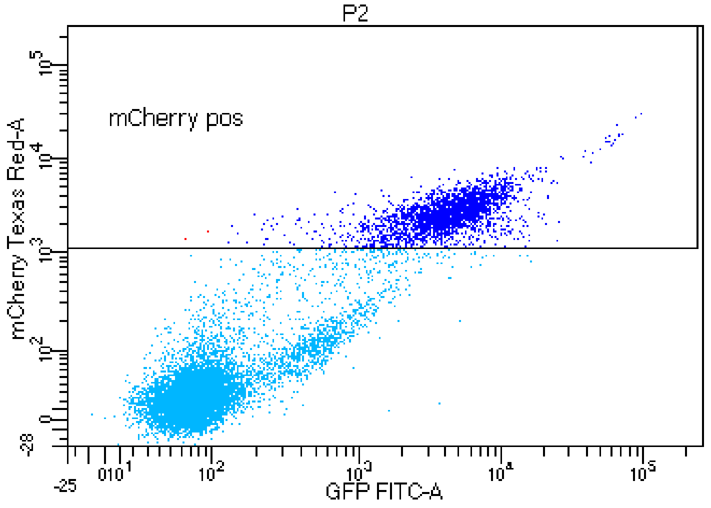

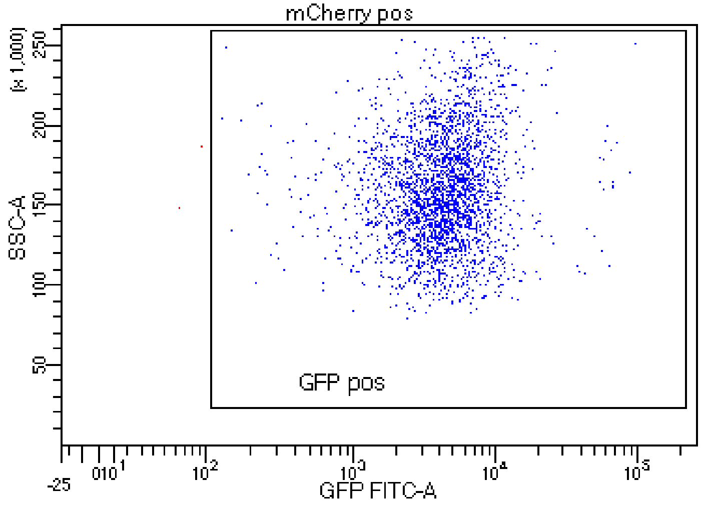

Tube: 2

Population	#Events	%Parent	%Total
All Events	17,151	####	100.0
P1	14,578	85.0	85.0
P2	14,346	98.4	83.6
mCherry pos	2,791	19.5	16.3
GFP pos	2,789	99.9	16.3

190806_LinaH

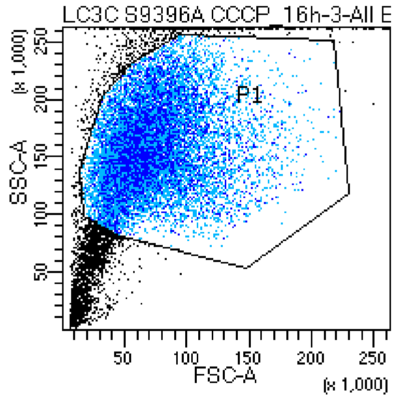

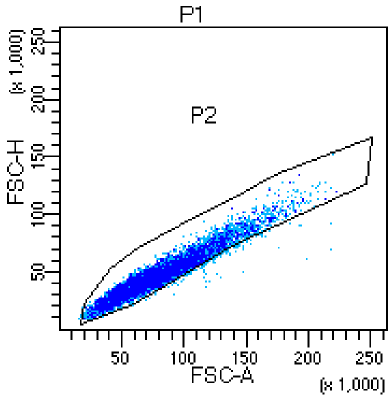

Experiment Name:	190806_LinaH
Specimen Name:	LC3C S9396A CCCP_16h
Tube Name:	3
Record Date:	Aug 6, 2019 10:27:49 AM

Population	#Events	%Parent	%Grand Pa...	mCherry T... Median	GFP FITC-A Median
All Events	17,642	####	####	32	91
P1	14,657	83.1	####	30	87
P2	14,385	98.1	81.5	30	86
mCherry pos	2,707	18.8	18.5	2,436	3,756
GFP pos	2,707	100.0	18.8	2,436	3,756

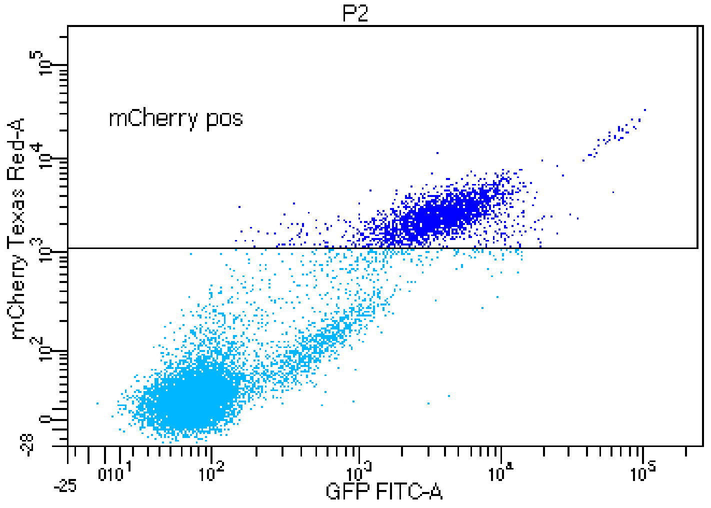

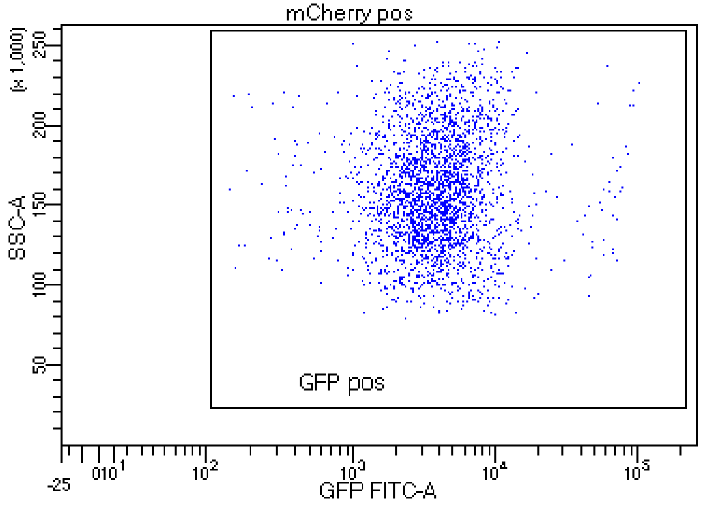

Tube: 3

Population	#Events	%Parent	%Total
All Events	17,642	####	100.0
P1	14,657	83.1	83.1
P2	14,385	98.1	81.5
mCherry pos	2,707	18.8	15.3
GFP pos	2,707	100.0	15.3

190806_LinaH

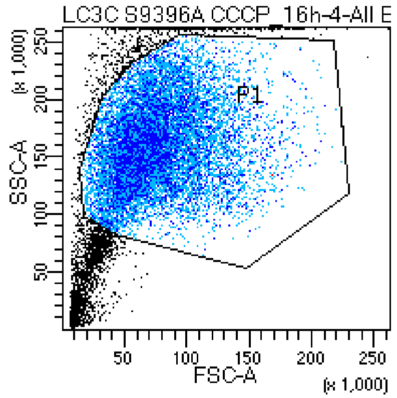

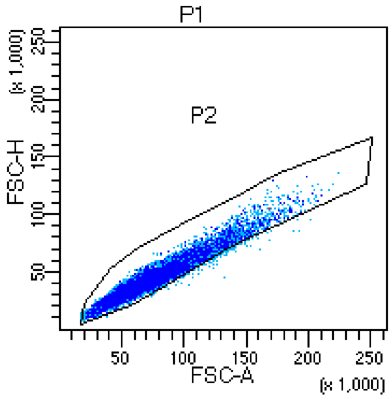

Experiment Name:	190806_LinaH
Specimen Name:	LC3C S9396A CCCP_16h
Tube Name:	4
Record Date:	Aug 6, 2019 10:29:20 AM

Population	#Events	%Parent	%Grand Pa...	mCherry T... Median	GFP FITC-A Median
All Events	16,547	####	####	30	86
P1	14,023	84.7	####	29	83
P2	13,756	98.1	83.1	29	82
mCherry pos	2,663	19.4	19.0	2,517	4,235
GFP pos	2,660	99.9	19.3	2,517	4,239

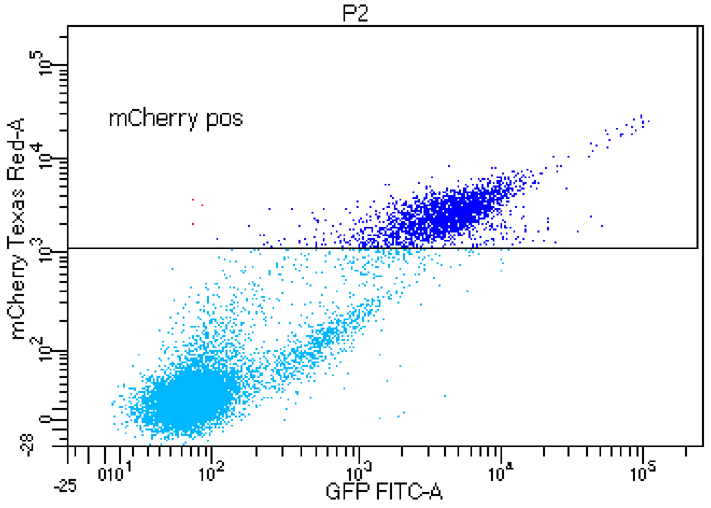

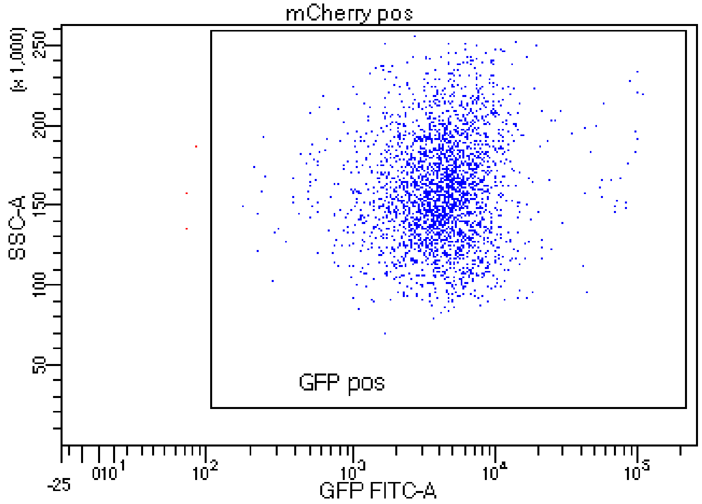

Tube: 4

Population	#Events	%Parent	%Total
All Events	16,547	####	100.0
P1	14,023	84.7	84.7
P2	13,756	98.1	83.1
mCherry pos	2,663	19.4	16.1
GFP pos	2,660	99.9	16.1

190806_LinaH

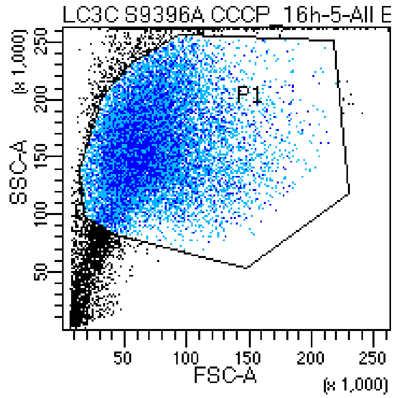

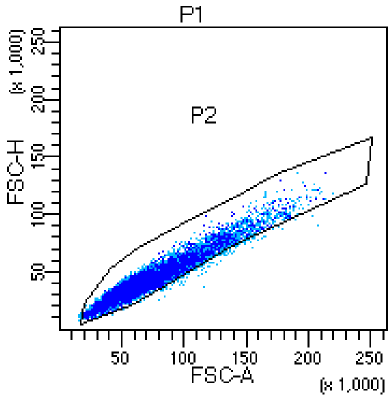

Experiment Name:	190806_LinaH
Specimen Name:	LC3C S9396A CCCP_16h
Tube Name:	5
Record Date:	Aug 6, 2019 10:31:08 AM

Population	#Events	%Parent	%Grand Pa...	mCherry T... Median	GFP FITC-A Median
All Events	19,244	####	####	31	88
P1	16,084	83.6	####	30	85
P2	15,813	98.3	82.2	29	84
mCherry pos	2,954	18.7	18.4	2,428	3,581
GFP pos	2,954	100.0	18.7	2,428	3,581

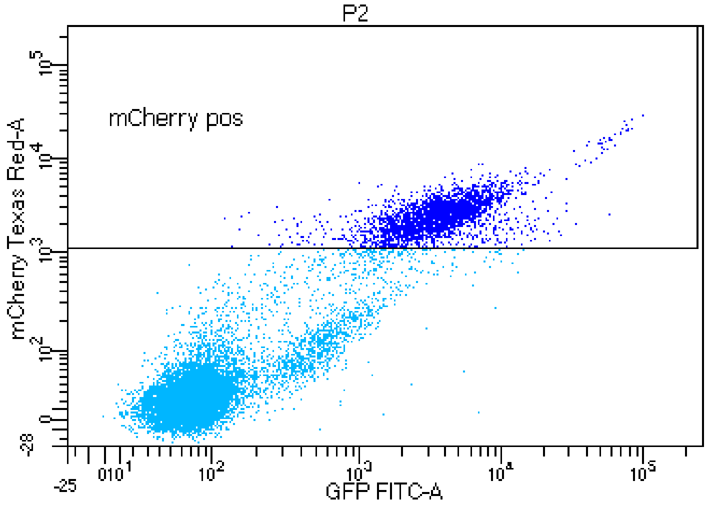

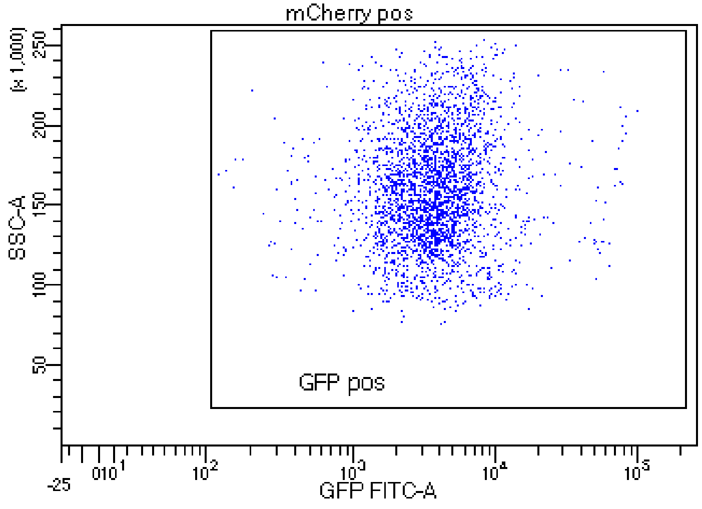

Tube: 5

Population	#Events	%Parent	%Total
All Events	19,244	####	100.0
P1	16,084	83.6	83.6
P2	15,813	98.3	82.2
mCherry pos	2,954	18.7	15.4
GFP pos	2,954	100.0	15.4

190806_LinaH

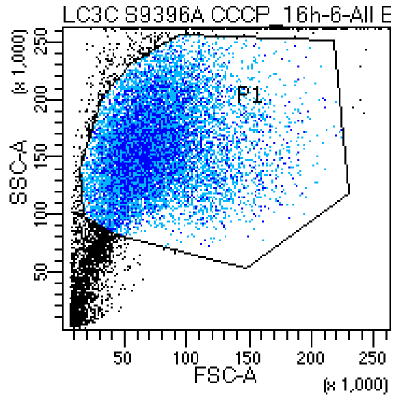

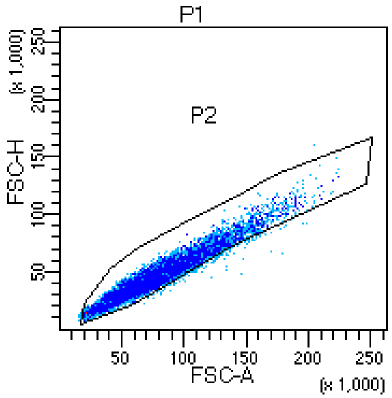

Experiment Name:	190806_LinaH
Specimen Name:	LC3C S9396A CCCP_16h
Tube Name:	6
Record Date:	Aug 6, 2019 10:32:55 AM

Population	#Events	%Parent	%Grand Pa...	mCherry T... Median	GFP FITC-A Median
All Events	17,376	####	####	31	90
P1	14,004	80.6	####	31	87
P2	13,708	97.9	78.9	30	86
mCherry pos	2,612	19.1	18.7	2,472	3,899
GFP pos	2,611	100.0	19.0	2,472	3,899

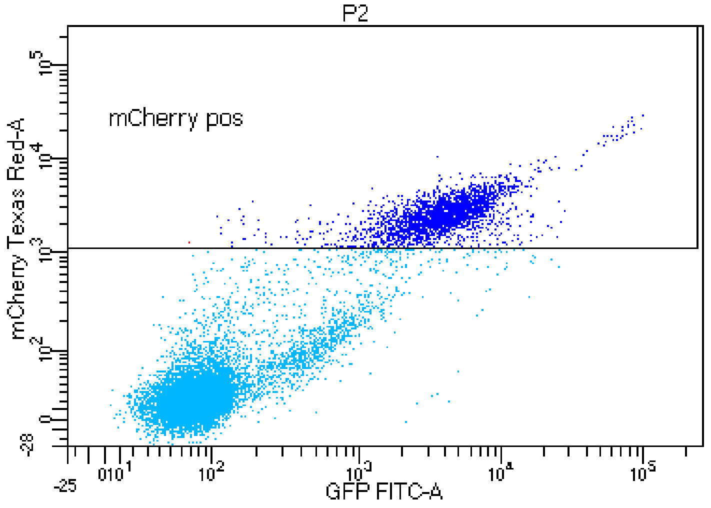

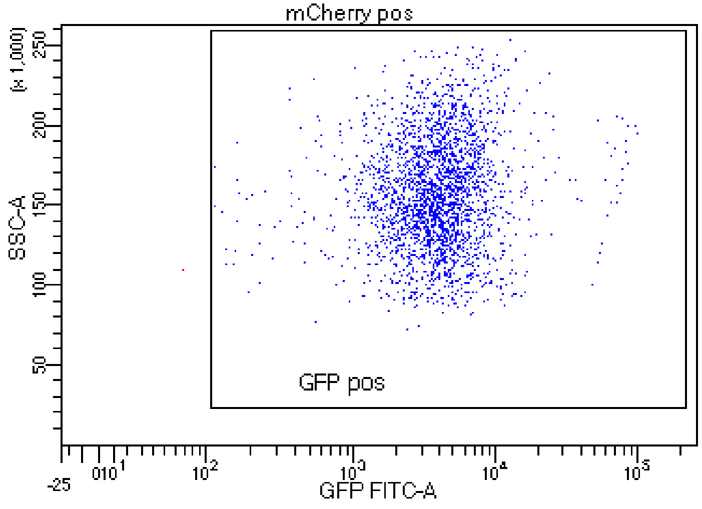

Tube: 6

Population	#Events	%Parent	%Total
All Events	17,376	####	100.0
P1	14,004	80.6	80.6
P2	13,708	97.9	78.9
mCherry pos	2,612	19.1	15.0
GFP pos	2,611	100.0	15.0
